# Switching p-type to high-performance n-type organic electrochemical transistors via doped state engineering

**DOI:** 10.1038/s41467-022-33553-w

**Published:** 2022-10-10

**Authors:** Peiyun Li, Junwei Shi, Yuqiu Lei, Zhen Huang, Ting Lei

**Affiliations:** 1 Key Laboratory of Polymer Chemistry and Physics of Ministry of Education, School of Materials Science and Engineering, Peking University, Beijing, 100871 China; 2 College of Chemistry and Molecular Engineering, Peking University, Beijing, 100871 China; 3 College of Engineering, Peking University, Beijing, 100871 China

**Keywords:** Electronic devices, Electronic materials

## Abstract

High-performance n-type organic electrochemical transistors (OECTs) are essential for logic circuits and sensors. However, the performances of n-type OECTs lag far behind that of p-type ones. Conventional wisdom posits that the LUMO energy level dictates the n-type performance. Herein, we show that engineering the doped state is more critical for n-type OECT polymers. By balancing more charges to the donor moiety, we could effectively switch a p-type polymer to high-performance n-type material. Based on this concept, the polymer, P(gTDPP2FT), exhibits a record high n-type OECT performance with μC* of 54.8 F cm^−1^ V^−1^ s^−1^, mobility of 0.35 cm^2^ V^−1^ s^−1^, and response speed of *τ*_on_/*τ*_off_ = 1.75/0.15 ms. Calculations and comparison studies show that the conversion is primarily due to the more uniform charges, stabilized negative polaron, enhanced conformation, and backbone planarity at negatively charged states. Our work highlights the critical role of understanding and engineering polymers’ doped states.

## Abstract (web summary)

Conventional strategies to obtain n-type organic electrochemical transistors are based on lowering the lowest unoccupied molecular orbital. Here Lei et al., engineer the polymer doped states to fabricate high-performance n-type organic electrochemical transistors.

## Introduction

Organic electrochemical transistors (OECTs) have attracted increasing interest because they have shown broad applications in neural interfacing devices, biochemical sensors, and neuromorphic computing applications^1,2^. Various p-type polymers have been developed for high-performance OECTs with their figure of merit, μC*, beyond 200 F cm^−1^ V^−1^ s^−1^ ^3–5^. These p-type polymers also exhibit fast response speed with the *τ*_on_/*τ*_off_ less than 1/0.1 ms, which are beneficial for real-time high-speed sensing applications^4^. To build complementary logic circuits for realizing high sensitivity and multiple device functions, n-type OECTs with comparable performance are necessary^6–8^. Unfortunately, compared to p-type ones, n-type OECT materials lag far behind in terms of both quantity and device performance, with μC* usually less than 1 F cm^−1^ V^−1^ s^−1^ and *τ*_on_/*τ*_off_ over 10 ms^9–16^.

In the development of n-type organic field-effect transistor (OFET) materials, researchers usually introduce more electron-deficient moieties to lower the lowest unoccupied molecular orbital (LUMO) energy level. This “lowering LUMO” strategy effectively enhances electron mobility and has promoted the fast development of n-type OFETs^16–24^. Inspired by this strategy, in the last 3 years, several strong electron-deficient n-type building blocks have been designed and used for OECTs, including naphthalene diimide (NDI)^9,11,25^, benzodifurandione-based oligo(*p*-phenylene vinylene) (BDOPV)^26^, bithiophene imide (BTI)^27,28^, pyrazine-flanked diketopyrrolopyrrole (PzDPP)^11^, 7,7′-diazaisoindigo (AIG)^29^, and some ladder-type polymers^15,30^. The μC* of the n-type OECT materials have been greatly enhanced from less than 0.1 F cm^−1^ V^−1^ s^−1^ to over 10 F cm^−1^ V^−1^ s^−1^ ^9,28^. However, most of these works require the synthesis of complicated acceptor moieties with lengthy and expensive synthetic steps, which impedes the practical applications of n-type OECTs. P-type materials, such as p(g2T2-g4T2) and pgBTTT, have not only simple molecular structures and short synthetic steps but also exhibit good performances^5,31^. Such considerable disparity makes us wonder whether there is a simple but efficient approach to high-performance n-type OECT materials.

**Fig. 1:**
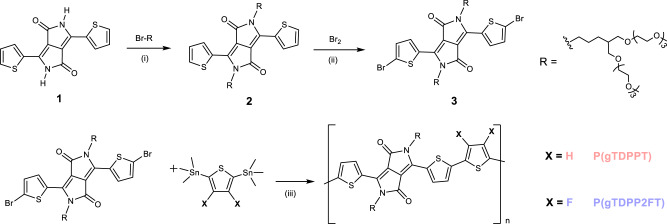
Synthetic routes to the polymers, P(gTDPPT) and P(gTDPP2FT). Reagents and conditions: (i) K_2_CO_3_, DMF, 110 °C, 12 h; (ii) Br_2_, DCM, 0 °C, 2 h; (iii) Pd(PPh_3_)_4_, CuI, Toluene/NMP (v/v = 1/1), 110 °C, 48 h.

Unlike the interface doping characteristics in OFETs, the whole polymer films in OECTs are highly doped by electrolytes during operation^32,33^. We propose that the molecular properties at the neutral state cannot simply determine the charge carrier transport characteristics during OECTs operation; however, the electronic structures and properties at the doped state may play a decisive role. Here, we choose thiophene-flanked diketopyrrolopyrrole (TDPP), one of the most simple and commercially available building blocks for study. To date, all the OECT materials based on TDPP are p-type or ambipolar^34–36^. We found that the introduction of two fluorine atoms on thiophene donors makes p-type P(gTDPPT) switch to pure n-type P(gTDPP2FT) (Fig. 1). P(gTDPP2FT) exhibits a record-high n-type OECT performance with μC* of 54.8 F cm^−1^ V^−1^ s^−1^. The polymer also shows record-high electron mobility of 0.35 cm^2^ V^−1^ s^−1^ in water, with a fast response speed of *τ*_on_/*τ*_off_ = 1.75/0.15 ms, which are among the shortest response times in n-type OECTs. Through theoretical and experimental exploration, we reveal that besides lowering the LUMO energy level, tuning the electronic properties at the polymer doped state results in more uniform charge distributions, enhanced backbone planarity, better conformation, and polaron stability, and finally leads to higher n-type OECT performance.

## Results

### Polymer synthesis and characterization

Commercially available TDPP (**1**) was used as the acceptor moiety. Compared with other n-type materials with long synthetic steps, the bromo-substituted TDPP (**3**) monomer used for polymerization only needs two steps to be obtained^11,15,25,36,37^. To compare the different electronic structures of the doped states, 2,5-bis(trimethylstannyl)thiophene (T) and 2,5-bis(trimethylstannyl)−3,4-difluorothiophene (2FT) were chosen to construct two similar polymers, namely P(gTDPPT) and P(gTDPP2FT) (Fig. 1). Ethylene glycol side chains (R) with farther branched positions were chosen for a closer π−π stacking distance and potentially enhanced charge carrier mobility, as we reported before^38,39^. Both polymers were obtained via Pd-catalyzed Stille coupling reactions in the presence of CuI as the co-catalyst^40^. Both polymers were purified by Soxhlet extraction and finally collected by chloroform. The molecular weights of the polymers were evaluated by gel permeation chromatography (GPC) using hexafluoroisopropanol (HFIP) as the eluent, *M*_w_/*M*_n_ = 67.4/32.6 kDa for P(gTDPPT) and *M*_w_/*M*_n_ = 65.0/30.7 kDa for P(gTDPP2FT), comparable to other p- or n-type polymers^4^ (Fig. S1). Both polymers exhibit good thermal stability with high decomposition temperatures (Figs. S2 and S3).

**Fig. 2:**
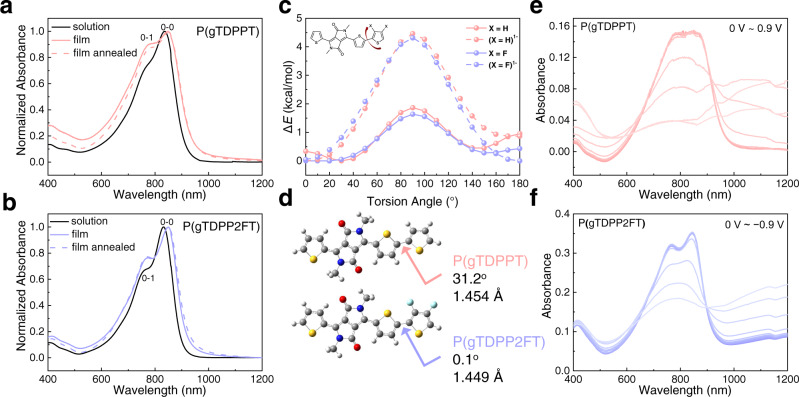
Optoelectronic properties of both polymers. Normalized UV-vis-NIR absorption spectra of **a** P(gTDPPT) and **b** P(TDPP2FT) in chlorobenzene (CB) solution, in a thin film, and annealed film on glass (80 °C, 10 min). **c** Comparison of the relaxed PES scans of the dihedral angles for the monomers of P(gTDPPT) and P(gTDPP2FT) in the neutral and negatively charged states. **d** Optimized backbone structures, bond lengths, and dihedral angles for the monomers. Electrochemical absorption spectra of **e** P(gTDPPT) and **f** P(TDPP2FT) on ITO glass in 0.1 M NaCl aqueous solution.

The optoelectronic properties of both polymers were evaluated using UV-Vis-NIR absorption spectra, cyclic voltammetry (CV), and spectroelectrochemistry (Fig. 2). The spectrum of P(gTDPP2FT) shows very similar maximum absorption peak (832 nm) and bandgap (1.34 eV) to that of P(gTDPPT) (836 nm, 1.36 eV) (Fig. 2a, b). Both spectra of P(gTDPPT) and P(gTDPP2FT) show a redshift in film and annealed film compared with the solution state, largely due to the further aggregation of the polymers. The 0-0/0-1 vibrational absorption peak ratio of P(gTDPP2FT) is larger than that of P(gTDPPT), suggesting a more planar backbone structure^41^. The spectra results are consistent with the relaxed potential energy surface (PES) scan calculations. The PES scans at the dihedral angles of the TDPP-T/2FT show that both polymers have similar torsional barriers. P(gTDPP2FT) exhibits a dominant conformation at 0^o^ at the TDPP-2FT dihedral angle, while (P(gTDPPT) exhibits a 30^o^ dihedral angle (Fig. 2c).The measured ionic potentials (IP) and electron affinities (EA) of P(gTDPP2FT) are estimated to be 5.20 and 3.86 eV, higher than that of P(gTDPPT) (4.86 and 3.69 eV) (Fig. S4a, b), which is consistent with the DFT calculation results (Table S1 and Fig. S5). Continuous CV sweep measurements of two polymers were explored in 0.1 M NaCl aqueous solution as the electrolyte, and both show good electrochemical stability (Fig. S4c, d). Interestingly, the DFT calculated torsion barriers of both polymers increase further after being n-doped. Besides, the bond length of TDPP2FT (1.449 Å) is shorter than TDPP-T (1.454 Å), suggesting the enhanced conjugation of P(gTDPP2FT) (Fig. 2d).

Spectroelectrochemistry was performed to investigate the electrochemical characteristics of both polymers (Fig. 2e, f). Since P(gTDPPT) is a p-type OECT material, it was charged by oxidization, while P(gTDPP2FT) was charged by reduction. Driven by the positive voltage, chloride ions in the electrolyte penetrate into the P(gTDPPT) film to keep the charge neutrality of the polymer film. On the contrary, P(gTDPP2FT) was reduced by the negative voltage, and sodium ions are the counter ions. The electrochemical doping process generated polarons/bipolarons in both polymer films, showing absorption bands in the long-wavelength region. The neutral polymers’ absorption bands (700–900 nm) decrease, and the polaron/bipolaron absorption bands (900–1200 nm) rise. The 0-0/0-1 vibrational absorption peak ratio (A_0-0_/A_0-1_) of P(gTDPPT) decreased close to 1 when immersed in NaCl aqueous solution (Fig. 2e). We found that A_0-0_/A_0-1_ is almost unchanged after exposure and reduction in electrolyte for P(gTDPP2FT), while A_0-0_/A_0-1_ decreased significantly for P(gTDPPT) (Fig. S6). These results indicate that P(gTDPP2FT) is more planar and conformationally stable than P(gTDPPT) during electrochemical operation.

### OECT device fabrication and characterization

The OECT devices were fabricated using photolithography and parylene patterning method^35^. The polymers were deposited using their chlorobenzene solution by spin-coating (see SI for more details). To evaluate the performance of an OECT material, the following equation based on the Bernards’ model is often used (Eq. 1)^42^:

1 $${g}_{{{{{{\rm{m}}}}}}}=(W/L)\cdot d\cdot \mu \cdot {C}^{*}\cdot {{{{{\rm{|}}}}}}({V}_{{{{{{\rm{th}}}}}}}-{V}_{{{{{{\rm{GS}}}}}}}){{{{{\rm{|}}}}}}$$

where *g*_m_ is the transconductance in the saturation regime, *W*, *L*, and *d* are the channel width, length, and film thickness, respectively, *μ* denotes the charge carrier mobility, C* denotes the capacitance of the channel per unit volume, *V*_th_ is the threshold voltage, and *V*_GS_ is the voltage between gate and source electrode.

**Fig. 3:**
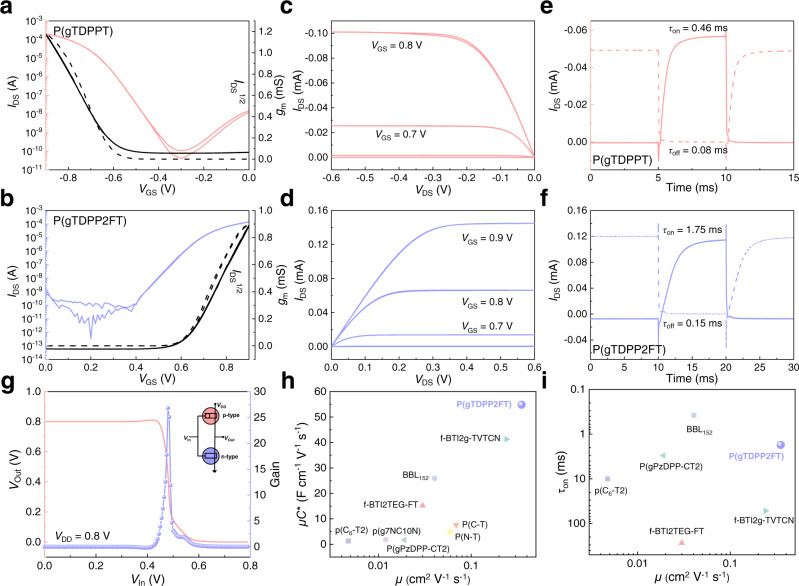
OECT device characterization of P(gTDPPT) and P(gTDPP2FT). **a**, **b** Transfer characteristics and **c**, **d** output characteristics of P(gTDPPT) and P(gTDPP2FT). The dash lines are the curve of *g*_m_. **e**, **f** Transient on/off curves with *V*_GS_ of 0~−0.9 and 0–0.9 V for P(gTDPPT) and P(gTDPP2FT), respectively. Device configuration: *W/L* = 100/10 μm, |*V*_DS_ | = 0.6 V. **g** Voltage transfer characteristics and gain of the complementary inverter based on P(gTDPPT) and P(gTDPP2FT). Insert is the circuit diagram of the complementary inverter. Device configuration: *W/L* = 100/10 μm. Comparison of the **h** μC* and *μ*, and **i** *τ*_on_ and *μ* values of P(gTDPP2FT) with other reported n-type OECT materials^25, 27–30,38,45^.

We applied both positive and negative gate voltages for both polymer devices. P(gTDPPT) shows typical p-type OECT behaviors, with a μC* of up to 65.1 F cm^−1^ V^−1^ s^−1^, while P(gTDPP2FT) shows satisfactory pure n-type OECT behaviors, with a high μC* of up to 54.8 F cm^−1^ V^−1^ s^−1^ (Fig. 3a–d and Table 1), which is a record value in the literature reported to date. Both polymers show a similar threshold voltage with an absolute value of around 0.6 V. To exclude the potential side-chain effects, we also synthesized P(lgTDPP2FT) with the same backbone as P(gTDPP2FT) but a linear side chain (Fig. S7). P(lgTDPP2FT) also shows n-type OECT behaviors with a high μC* of 20.4 **±** 1.0 F cm^−1^ V^−1^ s^−1^, which demonstrates the high n-type OECT performance of P(gTDPP2FT) come from the introduction of F atoms, not the side chains. The volumetric capacitance (C*) was measured by electrochemical impedance spectrum (EIS) (Fig. S8). The maximal C* was extracted with an average value of 161 F cm^−3^ for P(gTDPPT) and 156 F cm^−3^ for P(gTDPP2FT). Based on the μC* and C* values, the hole/electron mobility (*μ*) was calculated to be 0.40 cm^2^ V^−1^ s^−1^ for P(gTDPPT) and 0.35 cm^2^ V^−1^ s^−1^ for P(gTDPP2FT). Furthermore, we tested the transient characteristics of their OECT devices to evaluate the response speed of both polymers (Fig. 3e, f). A pulse was applied to the gate electrode, and a DC voltage with an absolute value of 0.6 V was applied to the drain electrode. The response time was estimated by an exponential fitting of the *I*_DS_. P(gTDPPT) and P(gDPP2FT) both exhibit short response times, with *τ*_on_/*τ*_off_ of 0.46/0.08 ms and 1.75/0.15 ms, respectively. The high *μ* and fast response characteristics make P(gTDPP2FT) a promising material for real-time high-speed sensing applications. The polymer also shows good stability with current retention of 54% after 3600 s on-off cycling measurement in the air (Fig. S9a). The device shows nearly unchanged performances after storing in the air for 1 month (Fig. S9b). The devices exhibit normal OECT behaviors, as the charge carriers will gradually disappear after the removal of *V*_GS_ (Fig. S9c)^43^. We believe that by introducing traps or blocks for ions, P(gTDPP2FT) can also show non-volatile behaviors with a long charge retention time after removing *V*_GS_, which could be used for neuromorphic computing^44^. Besides, the absorption spectra of polymer film also show good stability during the reduction cycling test (retention of 95.2% after 400 cycles) (Fig. S9d, e). The two polymers were used to fabricate complementary inverters because of their matched operating voltage and device performance. When the supply voltage (*V*_DD_) is set to 0.8 V and the input voltage (*V*_In_) is swept from 0 to 0.8 V, a relatively high gain value (∂*V*_Out_/∂*V*_In_) of 26.8 was obtained (Fig. 3g). The μC* and *μ* of P(gTDPP2FT) are both record values, and the response times are among the shortest in n-type OECT materials (Fig. 3h, I and Table S3)^25,27–30,38,45^.

**Table 1:** Summary of the OECTs performance of the polymers

	Type	*g*_m_^a^ (mS)	*d* (nm)	*V*_th_^b^ (V)	*I*_on_/*I*_off_	μC* ^c^ (F cm^−1^ V^−1^ s^−1^)	*μ*^d^ (cm^2^/V s)	C* (F cm^−3^)	*τ*_on_ (ms)	*τ*_off_ (ms)
P(gTDPPT)	p	1.18	60.5	−0.62 ± 0.02	5 × 10^6^	65.1 (45.9 ± 13.7)	0.40 (0.29 ± 0.09)	161 ± 15	0.46	0.08
P(gTDPP2FT)	n	0.93	60.6	0.64 ± 0.01	5 × 10^6^	54.8 (42.2 ± 6.5)	0.35 (0.27 ± 0.04)	156 ± 24	1.75	0.15

^a^The *W*/*L* of all the devices is 100/10 μm. All the OECT devices were operated in a 0.1 M NaCl aqueous solution.

^b^*V*_th_ was determined by extrapolating the corresponding *I*_DS_^1/2^ vs. *V*_GS_ plots.

^c^Four devices were tested and computed for each polymer. μC* was calculated according to Eq. (1). The data outside the brackets are maximal data, and the inside ones are the average.

^d^*μ* was calculated from the μC* and the measured volumetric capacitance C*. The data outside the brackets are maximal data, and the inside ones are the average.

### Film microstructure characterization

**Fig. 4:**
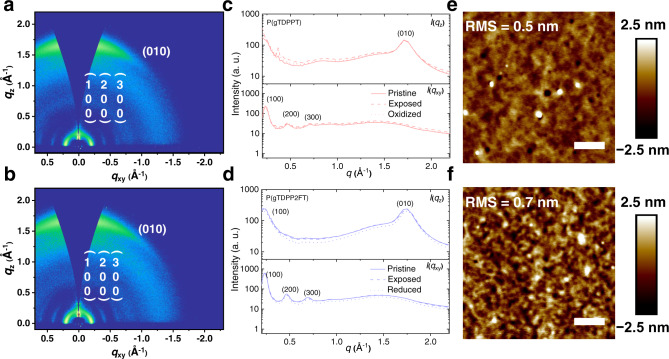
Molecular packing and morphology characterization. **a**, **b** 2D-GIWAXS patterns of P(gTDPPT) and P(gTDPP2FT). **c**, **d** The corresponding line cuts of the P(gTDPPT) and P(gTDPP2FT) GIWAXS pattern. Cuts along the *q*_xy_ direction represent scattering in the in-plane direction, while the *q*_z_ is from the out-of-plane direction. “Pristine” stands for dry films without any treatment. “Exposed” stands for the films immersed in 0.1 M NaCl for 10 min and blow-dried. “Oxidized/Reduced” stands for the films on the silicon substrate that are oxidized/reduced by a 0.9 V voltage bias for 10 min and blow-dried. **e**, **f** AFM height images of P(gTDPPT) and P(gTDPP2FT) films. The scale bars are 400 nm.

Grazing incidence wide-angle X-ray scattering (GIWAXS) and atomic force microscope (AFM) were employed to explore the molecular packing and morphology. Both P(gTDPPT) and P(gTDPP2FT) show typical face-on molecular packings with similar lamellar and π-π stacking distances (*d*_lamellar_ and *d*_π-π_) in their pristine films (Fig. 4a, b, Fig S10, and Table S4). After being exposed to 0.1 M NaCl aqueous solution, both polymers show an increase in *L*_c,lam_ (coherence length in lamellar direction), and a decrease in *L*_c,π-π_ (coherence length in π-π stacking direction). After oxidized, the *L*_c_ of P(gTDPPT) decreases significantly in both lamellar and π-π stacking directions, and the *g*_π-π_ (degree of paracrystalline disorder in π-π stacking) increases. However, for P(TDPP2FT), the *d*_lamellar_ remains unchanged, and the *d*_π-π_ decreases after exposure to water and reduction. After reduction, the *L*_c_ in both directions increases, and the *g*_π-π_ decreases compared to pristine (Fig. 4c, d and Table S4). These results prove that the molecular packing of P(TDPP2FT) is more ordered and stable after electrochemical doping, which is consistent with the above absorption spectra results (Fig. S6) and follow-up calculations. Both polymer films are smooth with small root-mean-square (RMS) roughness in atomic force microscope (AFM) height images (Fig. 4e, f). P(gTDPP2FT) film shows fiber-like textures, while P(gTDPPT) film is more amorphous.

### Understanding of the “doped state engineering” strategy

**Fig. 5:**
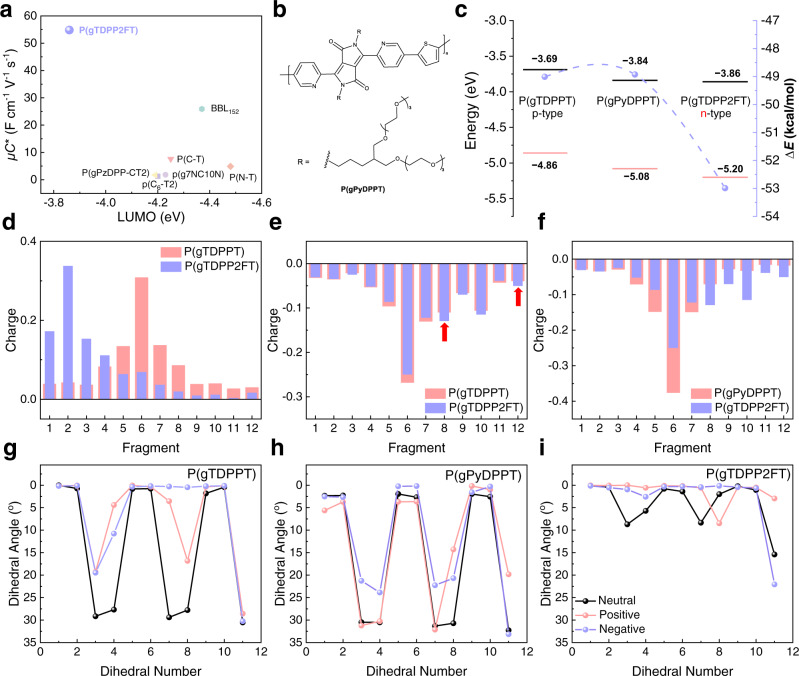
Understanding of the “doped state engineering”. **a** Comparison of the LUMO energy levels measured by CV and μC* of P(gTDPP2FT) and several reported n-type OECTs materials^25, 27–30,38,45^. **b** Chemical structure of the reference polymer, P(gPyDPPT). **c** Comparison of the HOMO/LUMO energy levels measured by the CV and the energy difference between neutral and negatively charged states (Δ*E* = *E*_negative_ − *E*_neutral_). Black lines stand for LUMO, and red lines stand for HOMO energy level. Purple dots stand for Δ*E* values. **d–f** Comparison of the charge distributions of the positively/negatively charged trimers of the three polymers. Comparison of **d** the positively and **e** the negatively charged P(gTDPPT) and P(gTDPP2FT). **f** Comparison of the negatively charged P(gPyDPPT) and P(gTDPP2FT). The charges of every fragment are the difference between the charged state and the neutral state. As shown by the red arrows, 2FT fragments in P(gTDPP2FT) shared more negative charges compared to the T fragments in P(gTDPPT). **g–i** The dihedral angle distribution between the T/D fragment and dihedral numbers are shown in Fig. S13.

The good OECTs performances of P(gTDPP2FT) are out of our expectations because its LUMO energy level is high compared with several typical n-type OECT materials (Fig. 5a). P(gPyDPPT), the thiophenes flanked to the DPP moiety of P(gTDPPT) are replaced by pyridines was also synthesized for comparison (Fig. 5b). P(gPyDPP-T2) with bithiophene as the donor moiety was reported by Giovannitti et al. It showed very poor p-type OECT performance^34^. We synthesized P(gPyDPPT) here, showing poor n-type OECT performance with μC* of 0.07 F cm^−1^ V^−1^ s^−1^ (Fig. S11). The introduction of pyridine and F atoms both reduce the LUMO energy level of P(gTDPPT) and the difference between the LUMO energy level of P(gPyDPPT) and P(gTDPP2FT) is less than 0.1 eV (Fig. 5c). We summarized the relationship between LUMO energy level and device performance of several n-type OECT polymers (Fig. 5a). The μC* value is not correlated well with LUMO energy levels. These results indicate that high-performance n-type OECT materials cannot be simply obtained by lowering the LUMO energy levels.

OECT materials usually work under highly doped states. We propose that the molecular properties under highly doped states might significantly affect the charge transport properties. Therefore, we calculated the properties of the three polymers’ doped states. The energy difference between the neutral and negatively charged state (Δ*E* = *E*_negative_ − *E*_neutral_) of P(gPyDPPT) is −48.93 kcal/mol, which is much smaller than that of P(gTDPP2FT) (−52.99 kcal/mol), and even smaller than that of P(gTDPPT) (−49.01 kcal/mol). These results suggest that the negative polarons on P(gTDPP2FT) backbone are more stable, and the stability is not related to the LUMO energy levels (Fig. 5a and Fig. S12). We also calculated the charge distribution of the three polymers relative to their neutral state (Fig. 5d–f and Fig. S13). Compared to [P(gTDPPT)]^1+^, the positive charges of [P(gTDPP2FT)]^1+^ are located on one end of the chain, suggesting that the positive charge might not be stable on P(gTDPP2FT) (Fig. 5d). Compared to [P(gTDPPT)]^1−^, the negative charges of [P(gTDPP2FT)]^1−^ are more delocalized, and the negative charges on the thiophene of DPP moieties are shared by the 2FT moieties (red arrows in Fig. 5e), making the charge distribution more balanced. The negative charges of [P(gTDPP2FT)]^1−^ distribute on the whole polymer chain, whereas the negative charges of [P(gPyDPPT)]^1−^ are primarily localized in the center of the chain (Fig. 5f). The more delocalized negative charge distribution may explain the better stability of the [P(gTDPP2FT)]^1−^. Besides, the distribution of dihedral angles between fragments is quite different for the three polymers (Fig. 5g–i). P(gTDPP2FT) shows the smallest dihedral angles along the polymer backbone at the neutral state, which decreases further after being negatively charged. Relatively large dihedral angles of P(gTDPPT) decrease a little after being negatively charged. Conversely, P(gPyDPPT) exhibits the largest dihedral angles, which do not change much after being both positively and negatively charged. All these charge and dihedral angle distributions prove that the introduction of fluorine atoms in P(gTDPP2FT) not only lowers the LUMO energy level but, more importantly, enhances the polymer backbone planarity, delocalizes, and stabilizes the negative polaron. The calculated results agree well with the above spectra and GIWAXS results (Fig. S6 and Fig. 4). These features might explain the good n-type charge transport behavior and high electron mobility of P(gTDPP2FT) under strong electrochemical n-doping. Although P(gTDPP2FT) exhibits good stability over cycling and storage, we do not deny the potential side reactions that relatively high LUMO levels may lead to, for example, oxygen reduction reaction (ORR). Therefore, in the future, in addition to our “doped state engineering”, further lowering the LUMO energy level is needed for n-type OECT materials.

## Discussion

In conclusion, we have proposed a “doped state engineering” strategy to design n-type OECT polymers and effectively switch a typical p-type OECT polymer to a high-performance n-type OECT polymer. We demonstrate that in addition to the lower LUMO energy level, the switching mechanism of the charge transport type is primarily due to the more uniform negative charge distribution, enhanced backbone planarity, better conformational stability, and more stable negative polaron after n-doping. These features make polymer P(gTDPP2FT) exhibit pure n-type charge transport behaviors with record-high electron mobility of 0.35 cm^2^ V^−1^ s^−1^ in water, record-high μC* values of 54.8 F cm^−1^ V^−1^ s^−1^, and a fast response speed of *τ*_on_/*τ*_off_ = 1.75/0.15 ms. Our work reveals the significant differences in the electronic properties between the charged and neutral states and highlights a “doped state engineering” strategy for future high-performance OECT materials design.

## Methods

### Materials

The synthesis and characterization of the polymers and the various synthetic intermediates are outlined in the Supplementary Information.

### Spectroelectrochemistry

Spectroelectrochemistry was performed with an ITO-coated glass slide, spun cast with the polymer solution (chlorobenzene (CB) solution). These polymer-coated ITO slides, a Pt mesh an Ag/AgCl pellet are immersed into the cuvette filled with 0.1 M aqueous NaCl solution. A PerkinElmer Lambda 750 UV-vis spectrometer was used with the beam path passing through the electrolyte-filled cuvette and polymer-coated ITO samples. The potential was applied for 5 s before the spectra were recorded and lasted for a certain amount of time until the completion of spectrum scanning.

### OECT fabrication and characterization

The OECTs fabrication included the deposition and patterning of the metallic electrodes, the parylene layer, and the polymer in the channel. In detail, silica substrates were cleaned by ultrasonication in acetone, DI water, and isopropyl alcohol, followed by nitrogen blow-drying and brief oxygen plasma cleaning. Metal pad interconnects and source/drain contacts were patterned and deposited (5 nm Cr and 35 nm Au) by lift-off methods. About 1 μm of parylene-C is deposited using a PDS 2010 Labcoater-2, with a 3-(trimethoxysilyl)propyl methacrylate (A-174 Silane) adhesion promoter. A 2% aqueous solution of industrial cleaner (Micro-90) was subsequently spun coated as an anti-adhesive for a second sacrificial 1 μm parylene-C film. Samples were subsequently patterned with AZ 10XT photoresist and AZ-400K developer. The patterned areas were etched by reactive ion etching with O_2_ plasma using an LCCP-6A reactive ion etcher (Leuven Instruments). The polymer was dissolved in chlorobenzene at a concentration of 3 mg/ml. The polymer solution was spin-cast on the etched devices. After a peeling-off process of the second sacrificial parylene layer, the OECTs were ready for measurement. The device characterization was performed on a probe station using a Keithley 4200 SCS analyzer or Fs-Pro semiconductor parameter analyzer, PDA. AgCl/Ag pellet (Warner Instruments) was employed as the gate electrode and immersed into a 0.1 M NaCl solution, which covers the polymer film in the channel. The thickness of the film was determined in a dry state after testing with a DEKTAK profilometer (Bruker).

### Electrochemical impedance spectra

Electrochemical impedance spectra (EIS) were performed on the polymer-coated electrodes using the electrochemical workstation SP-300 (BioLogic Science Instruments). Polymer film covered on the Au electrodes was patterned as squares with certain areas through the lithography technique. These polymer-coated Au electrodes with glass substrate were fully covered with a 0.1 M NaCl solution, followed by the employment of Pt mesh and Ag/AgCl pellet. The capacitances of polymers measured on Au electrodes of various sizes were obtained through the potential-EIS method, with setting the DC offset voltage as the maximum achievable doping for each polymer. The AC amplitude of voltage in the form of sine-wave on the WE was set as 10 mV (RMS) and the frequency was scanned from 1 Hz to 100 kHz. The as-obtained Bode plots or Nyquist plots were fitted to an equivalent circuit, namely the Randle’s circuit *R*_s_ (*R*_p_ | |*C*), via the software EC-Lab view. The thickness of the film was determined in a dry state after testing with a DEKTAK profilometer (Bruker).

### AFM and GIWAXS characterization

Atomic force microscopy (AFM) measurements were performed with Dimension icon ScanAsyst (Bruker). Two-dimensional grazing incidence wide-angle X-ray scattering(2D-GIWAXS) measurements were conducted on a Xenocs-SAXS/WAXS system with an X-ray wavelength of 1.5418 Å and 0.2° as an incidence angle. Pilatus 300 K was used as a 2D detector. Data processing was performed in Igor Pro software with Nika and WAXTools packages. Coherences length (*L*_c_) is calculated from the breadth (*Δq*) of a diffraction peak: *L*_c_ = 0.89 × 2π/*∆q*^46,47^. And paracrystalline disorder is calculated from the center position (*q*_0_) and breadth (*Δq*) of a diffraction peak: *g* = (*∆q*/(2π*q*_0_))^1/2^ ^46,47^.

## Supplementary information

Supplementary Information

## Data Availability

The source data generated in this study have been deposited in the materials cloud database (https://archive.materialscloud.org/record/2022.113) and are also available from the corresponding author upon request.

## Code availability

The codes that support the findings of this study are available from the corresponding author upon request.
