# Supplementary material for: Switching p-type to high-performance n-type organic electrochemical transistors via doped state engineering
Source: Nat Commun. 2022 Oct 10;13:5970. doi: 10.1038/s41467-022-33553-w (PMC9551099; doi:10.1038/s41467-022-33553-w)
Supplement: Supplementary file 1 — Supplementary Information [file 41467_2022_33553_MOESM1_ESM.pdf]

Supplementary Information

for

Switching p-type to high-performance n-type organic electrochemical transistors via doped state engineering

Peiyun Li[1,#], *Junwei Shi*[1,2,#], *Yuqiu Lei*[3], *Zhen Huang*[2], *and Ting Lei*[1,*]

[1]Key Laboratory of Polymer Chemistry and Physics of Ministry of Education, School of Materials Science and Engineering, Peking University, Beijing 100871, China

[2]College of Chemistry and Molecular Engineering, Peking University, Beijing 100871, China.

[3]College of Engineering, Peking University, Beijing 100871, China

[#]These authors contributed equally to this work

[*]E-mail: tinglei@pku.edu.cn

Table of Contents

1. Experimental Details

2. Supplementary Tables and Figures

3. Synthesis and Characterization of New Compounds

4. Supplementary References

1. Experimental Details

Materials

All the chemical reagents were purchased and used as received unless otherwise indicated. All air and water-sensitive reactions were performed under a nitrogen atmosphere. Dichloromethane (DCM), Tetrahydrofuran (THF), Toluene, and *N*, *N*-Dimethylformamide (DMF) were dried by a JC Meyer solvent drying system before use. Ultradry solvents were obtained from J&K reagent company.

Chemical structure and optoelectronic property characterization

^{1}H NMR and ^{13}C NMR spectra were recorded on Bruker ARX-400 (400 MHz). All chemical shifts were reported in parts per million (ppm). ^{1}H NMR chemical shifts were referenced to $CDCl_3$ (7.26 ppm), and ^{13}C NMR chemical shifts were referenced to $CDCl_3$ (77.16 ppm). Mass spectra were recorded on an FTMS Fourier transform high-resolution mass spectrometer. Thermal gravity analyses (TGA) were carried out on a TA Instrument Q600 SDT analyzer, and differential scanning calorimetry (DSC) analyses were performed on a TA Instrument Q2000 analyzer. Absorption spectra were recorded on PerkinElmer Lambda 750 UV-vis spectrometer. Cyclic voltammograms (CV) were measured through an electrochemical workstation SP-300 (BioLogic Science Instruments). A standard three-electrode setup was established by employing glassy carbon as the working electrode (WE), a block of platinum mesh as the counter electrode (CE), and an Ag/AgCl electrode as the reference electrode (RE), further calibrated against ferrocene (Fc/Fc^+). The measurements were carried out in an aqueous solution with 0.1 M NaCl or in acetonitrile with 0.1 M tetrabutylammonium hexafluorophosphate as the supporting electrolyte with a scan rate of 50 mV/s. Ionization potentials and electron affinity were obtained using the equation: $IP = (E_{Ox} - E_{Fc/Fc^+} + 4.8)$ eV, $EA = (E_{Red} - E_{Fc/Fc^+} + 4.8)$ eV.

Size exclusion chromatography measurement

Polymer number-average molecular weight (M_n) and molecular weight distributions

(PDI = M_w/M_n) were measured by size exclusion chromatography (SEC). HFIP SEC analyses were performed on a Waters 1515 instrument equipped with a PLMIXED 7.5 × 50 mm guard column, two PLMIXED-C 7.5×300 columns, and a differential refractive index detector at 35 °C with a flow rate of 1 ml min^{-1}. The instrument was calibrated with 10 PS standards, and chromatograms were processed with Waters Breeze software.

DFT calculations. Geometry optimization, molecular energy level calculation, and relaxed potential energy surface scan were performed at the B3LYP/6-311g(d,p) or wB97XD/6-311g(d,p) level using Gaussian 16 and Gaussian View 6[1,2]. The computational results were visualized by Multiwfn[3] and VMD[4].

2. Supplementary Tables and Figures

Table S1. Summary of the Energy Levels and Molecular Weights of Polymers

	**HOMO**[a] (eV)	**LUMO**[a] (eV)	**IP**[b] (eV)	**EA**[b] (eV)	E_b[c] (eV)	E_g[c] (eV)	M_n[d] (kDa)	**PDI**[d]
P(gTDPPT)	−4.92	−3.25	4.86	3.69	1.36	1.17	32.6	2.1
P(gTDPP2FT)	−5.03	−3.39	5.20	3.86	1.34	1.38	30.7	2.1

[a]From DFT calculations. [b]Estimated from the cyclic voltammetry (CV) measurement. [c]Optical band gap. Estimated from the UV-Vis-NIR spectra. [d]Using HFIP as the eluent.

Table S2. OECT Device Parameters for the Two Polymers

	W/L	g_m (mS)	d (nm)	V_{th} (V)	V_{GS} (V)	μC^* (F cm^{-1} V^{-1} s^{-1})
P(gTDPPT)	10	1.18	60.5	−0.60	−0.90	65.1
	10	0.78	60.5	−0.64	−0.86	53.7
	10	0.60	54.2	−0.60	−0.84	46.1
	10	0.20	54.2	−0.64	−0.84	18.5
Average				−0.62		45.9
P(gTDPP2FT)	10	0.52	60.6	0.64	0.89	34.3
	10	0.54	60.6	0.65	0.89	37.1
	10	0.67	60.6	0.64	0.90	42.5
	10	0.93	60.6	0.62	0.90	54.8
Average				0.64		42.2

Table S3. Comparison of the OECT Performances for the OECT Polymers reported in the literature.

Polymer	type	HOMO[a] (eV)	LUMO[a] (eV)	μ ($cm^2\ V^{-1}\ s^{-1}$)	μC^* ($F\ cm^{-1}\ V^{-1}\ s^{-1}$)	τ_{on} (ms)	Ref
P(C6-T2)	n	−5.6	−4.2	0.00474	1.29	10	5
BBL_{152}	n	−6.12	−4.37	0.0404	25.9	0.38	6
f-BTI2TEG-FT	n	−5.59	−3.82	0.0299	15.2	272	7
P(C-T)	n	−5.53	−4.25	0.069	7.6	NA	8
P(N-T)	n	−5.76	−4.48	0.059	4.9	NA	8
P(gPzDPP-CT2)	n	−5.72	−4.19	0.019	1.72	3	9
P(g7NC10N)	n	−5.12	−4.23	0.012	1.83	NA	10
f-BTI2g-TVTCN	n	−5.57	−3.81	0.24	41.3	52	11

[a]Estimated from the cyclic voltammetry (CV) measurement.

Table S4. Comparison of the Molecular Packing Parameters of Polymers under different conditions

		$d_{lamellar}$ (Å)	$d_{\pi\text{-}\pi}$ (Å)	$L_{c,lam}$ (Å)	$L_{c,\pi\text{-}\pi}$ (Å)	g_{lam}	$g_{\pi\text{-}\pi}$
P(TDPPT)	Pristine	26.5	3.64	47.3	19.7	0.20	0.16
	Exposed	26.9	3.64	52.9	19.2	0.19	0.16
	Oxidized	26.6	3.64	46.0	17.4	0.20	0.17
P(TDPP2FT)	Pristine	26.8	3.62	46.0	16.1	0.20	0.18
	Exposed	26.8	3.60	47.7	15.6	0.20	0.18
	Reduced	26.8	3.59	46.4	16.8	0.20	0.17

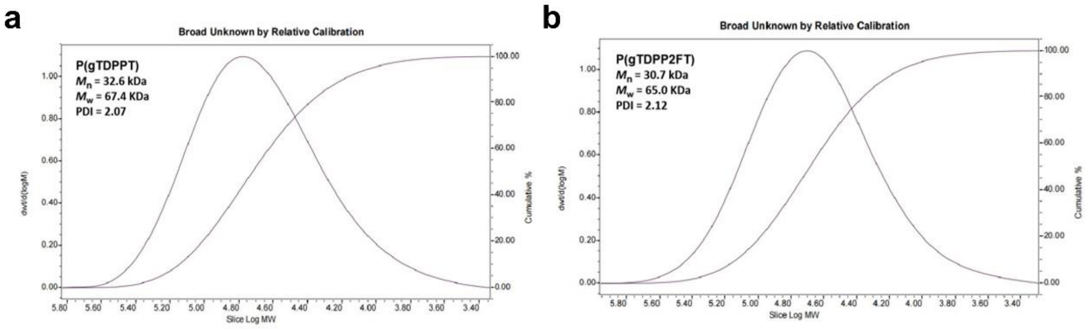

Fig. S1 Molecular weights and polymer dispersity index (PDI) of the polymers. **a** P(gTDPPT), **b** P(gTDPP2FT), measured by GPC with HFIP as the eluent.

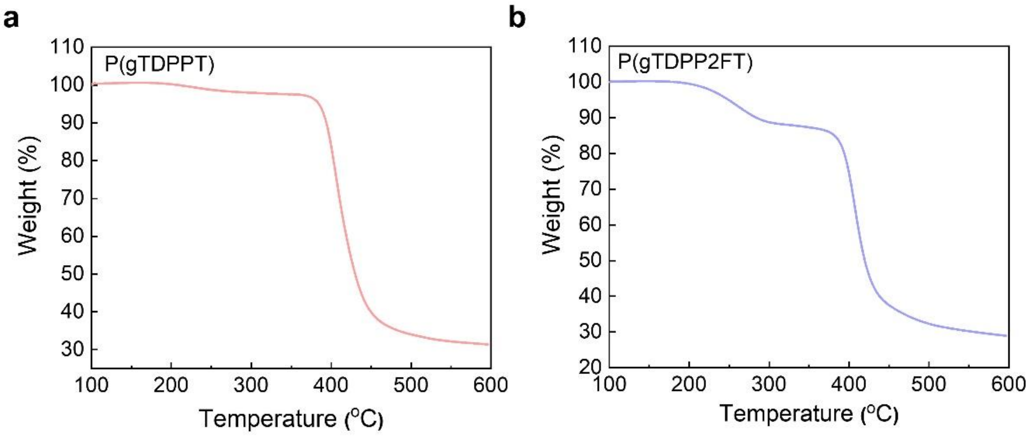

Fig. S2 Thermal gravity analysis (TGA) of the polymers. **a** P(gTDPPT), **b** P(gTDPP2FT). Both polymers showed high decomposition temperatures of over 200 °C

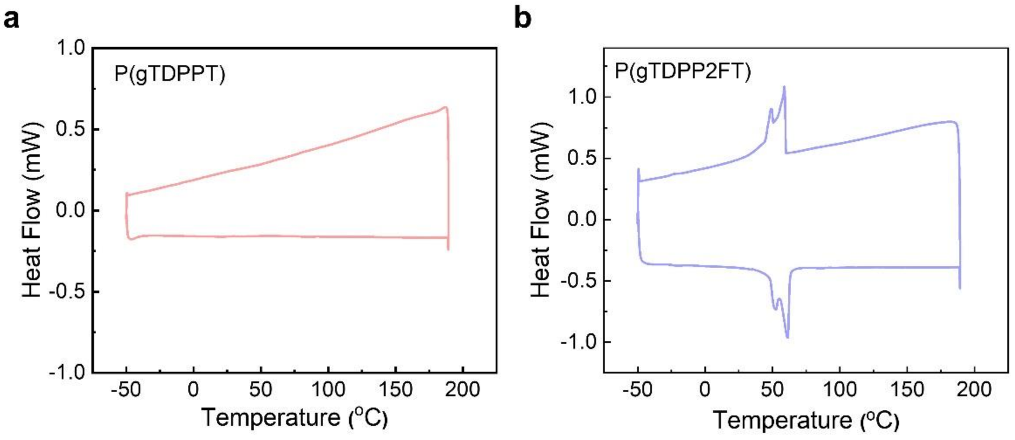

Fig. S3 Differential scanning calorimetry (DSC) of the polymers. a P(gTDPPT), **b** P(gTDPP2FT).

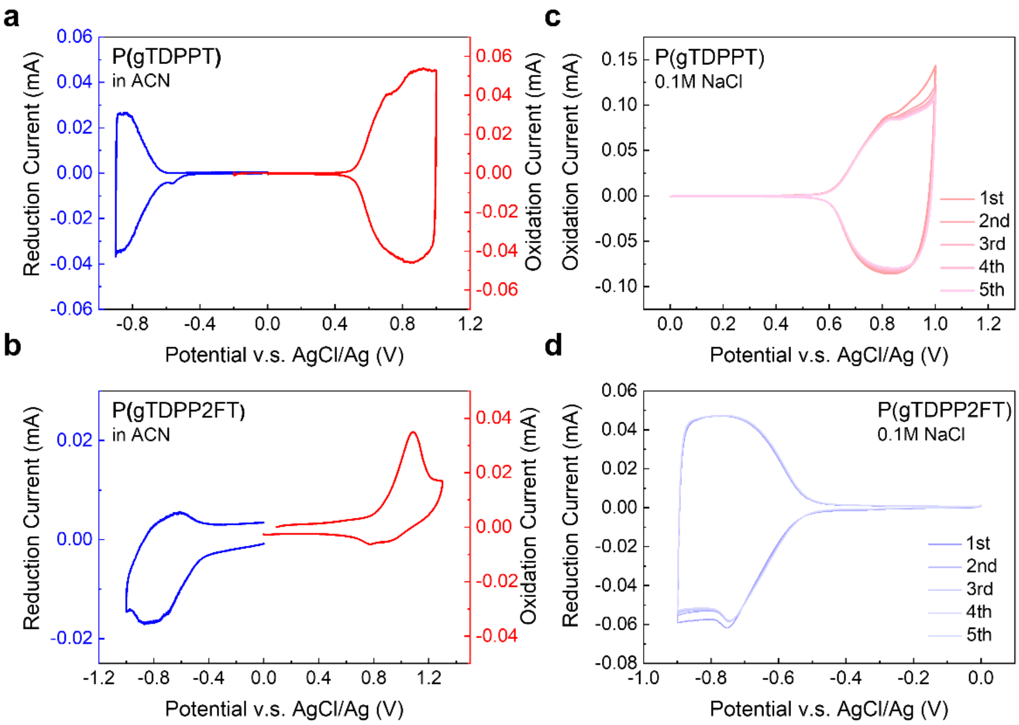

Fig. S4 Cyclic voltammograms of the polymers. **a & c** P(gTDPPT) and **b & d** P(gTDPP2FT), in acetonitrile solution with 0.1 M tetrabutylammonium hexafluorophosphate or 0.1 M NaCl as the electrolyte.

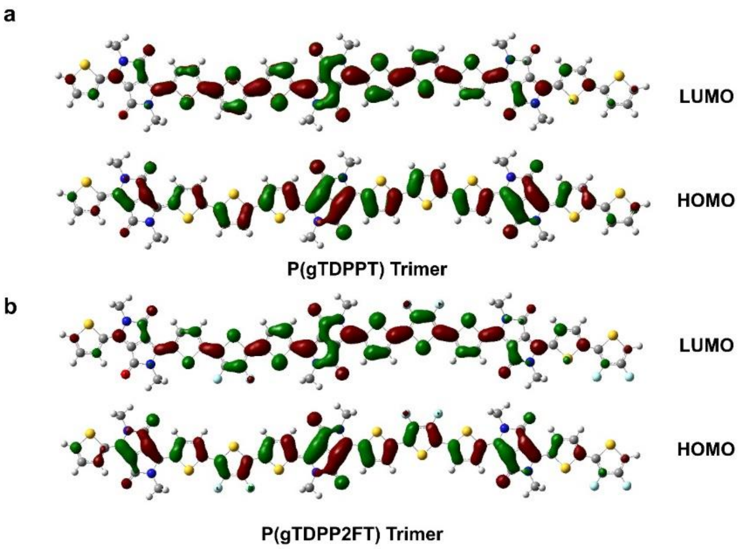

Fig. S5 DFT-optimized geometries and molecular frontier orbitals of the polymers. The trimers of **a** P(gTDPPT), **b** P(gTDPP2FT). Calculations were performed at B3LYP/6-311G(d,p) level. Branched glycol side chains were replaced with methyl groups to simplify the calculation.

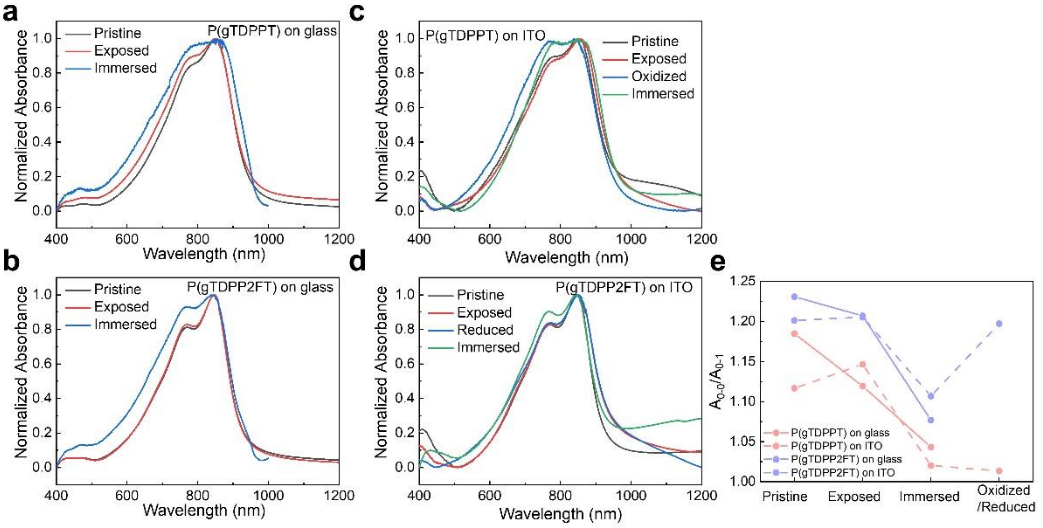

Fig. S6 Normalized UV-vis-NIR absorption spectra of P(gTDPPT) and P(gTDPP2FT) films. a & b Films on glass substrates. **c & d** Films on ITO-coated glass substrates. **e** 0-0/0-1 vibrational peak ratio of different conditions of polymer films. “Pristine” stands for the dry films without any treatment. “Exposed” stands for the films immersed in 0.1 M NaCl for 10 mins and blow-dried. “Immersed” stands for the films immersed in 0.1 M NaCl during the spectra test. “Oxidized/Reduced” stands for the films on the ITO-coated glass substrates that are oxidized/reduced by a 0.9 V voltage bias for 10 mins and blow-dried. Rayleigh scattering correction was performed on all spectra.

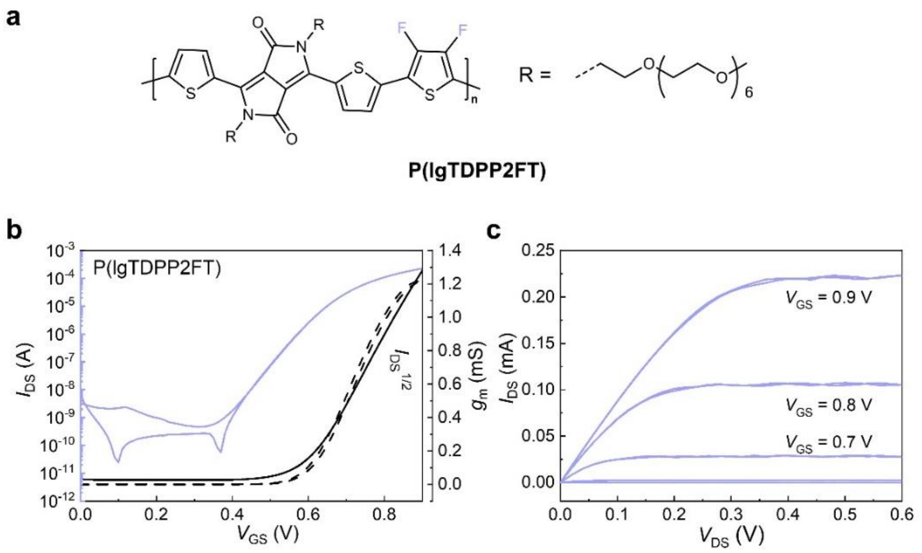

Fig. S7 Chemical structure and OECTs performance of P(lgTDPP2FT). a Chemical structure of P(lgTDPP2FT). **b & c** Transfer characteristics and output characteristics of P(lgTDPP2FT). *W/L* = 100/10 μm of all devices and V_{DS} = 0.6 V for Fig. b. The μC^* is 20.4±1.0 F cm^{-1} V^{-1} s^{-1}.

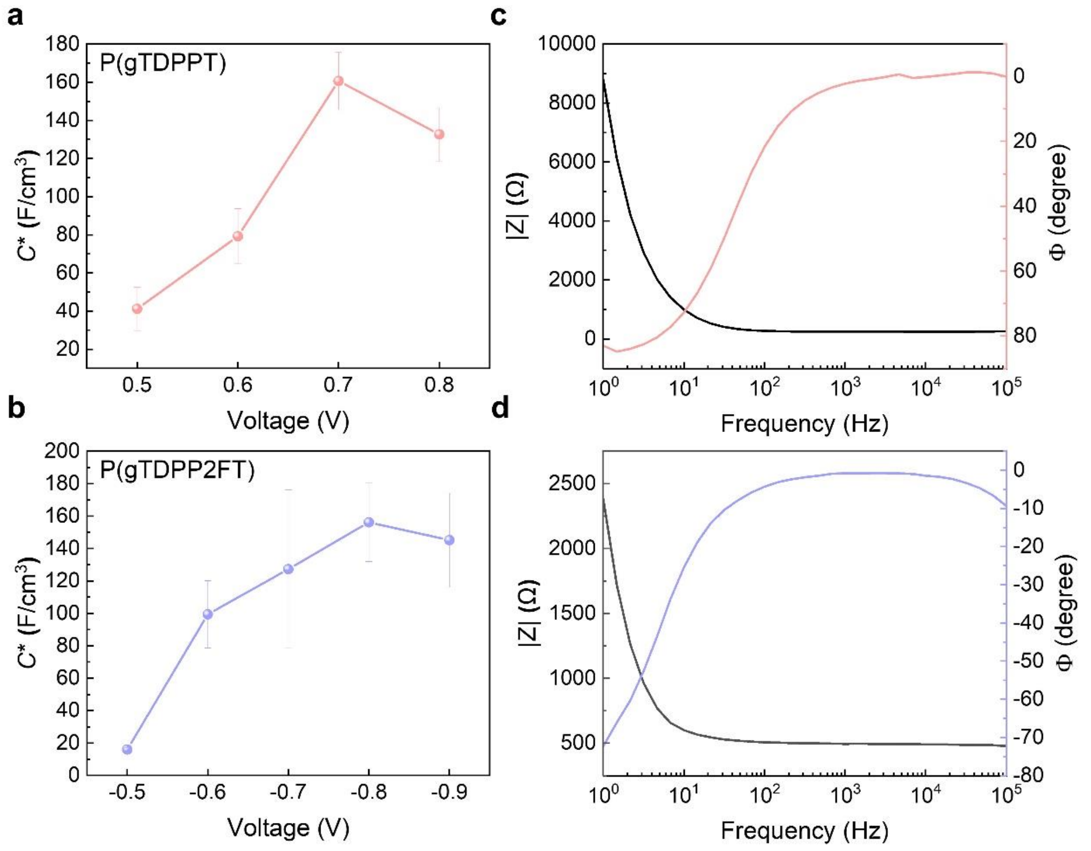

Fig. S8 Capacitive behaviors of both polymers. a & b Voltage-capacitance relationship of P(gTDPPT) and P(gTDPP2FT) measured through the electrochemical impedance spectrum. **c & d** The corresponding Bode and phase plot of P(gTDPPT) and P(gTDPP2FT) with a bias of 0.7 V and −0.8 V, respectively.

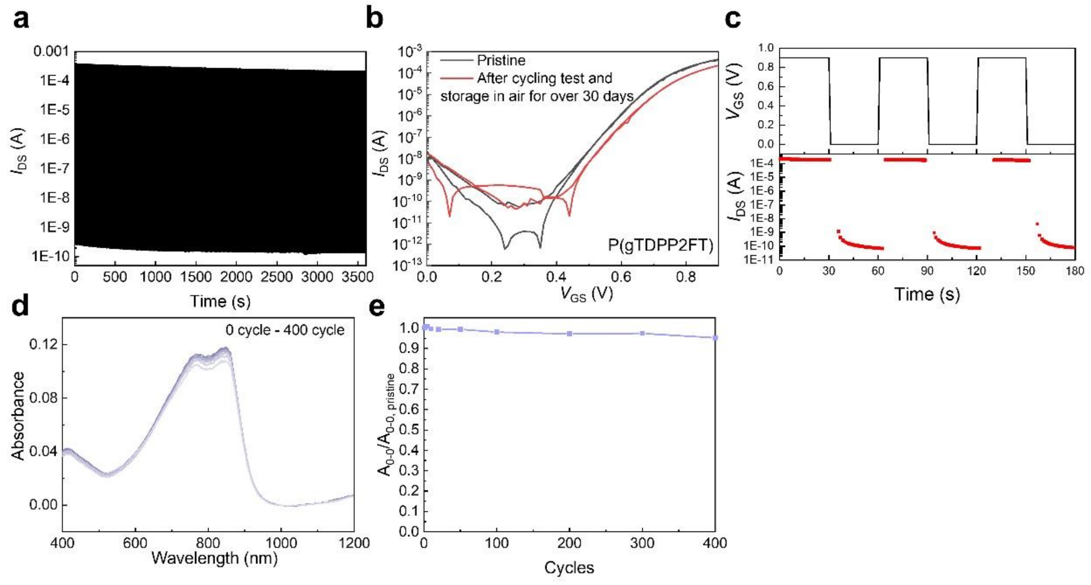

Fig. S9 Stability of P(gTDPP2FT). a Long-term on-off switching of P(gTDPP2FT) in 0.1 M NaCl

aqueous solution. **b** Transfer characteristics of P(gTDPP2FT) before and after long time cycling and storage. The current retention is over 50 %. V_{DS} = 0.6V, gate voltage pulse V_{GS} = 0.9 V, t_{on}/t_{off} = 7/7 s. W/L = 100/10 μm, d = 161.0 nm. **c** Charge retention measurement of P(gTDPP2FT). The V_{GS} is applied to the channel for 30 s and then removed for 30 s, cycling three times. **d** In situ UV-vis-NIR absorption spectra of P(gTDPP2FT) films. The spectrum is measured after every reduction cycle. The picture shows the pristine film and the spectra after the 1st, 2nd, 5th, 10th, 20th, 50th, 100th, 200th, 300th, and 400th reduction cycles. **e** Changes of the 0-0 peak absorption compared to the pristine film during the cycling test. The absorption spectra are almost unchanged (retention of 95.2%) over 400 reduction cycles. The results prove that P(gTDPP2FT) can maintain structural stability after reduction.

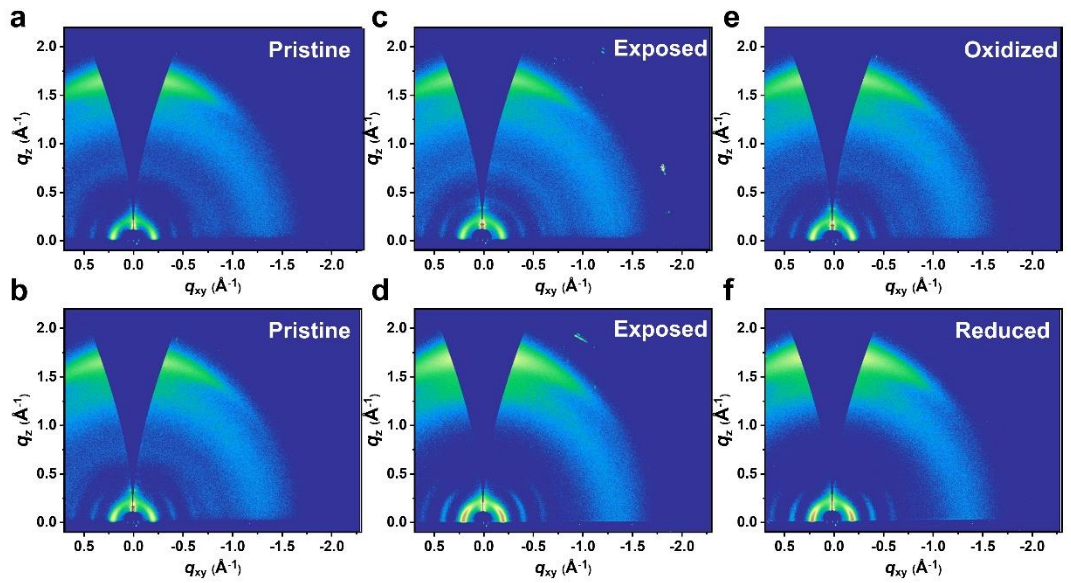

Fig. S10 2D-GIWAXS patterns of the polymers. The pristine, exposed and oxidized/reduced films of **a, c, & e** P(gTDPPT) and **b, d & f** P(gTDPP2FT), respectively. “Pristine” stands for the dry films without any treatment. “Exposed” stands for the films immersed in 0.1 M NaCl for 10 mins and blow-dried. “Oxidized/Reduced” stands for the films on the silicon substrate that are oxidized/reduced by a 0.9 V voltage bias for 10 mins and blow-dried.

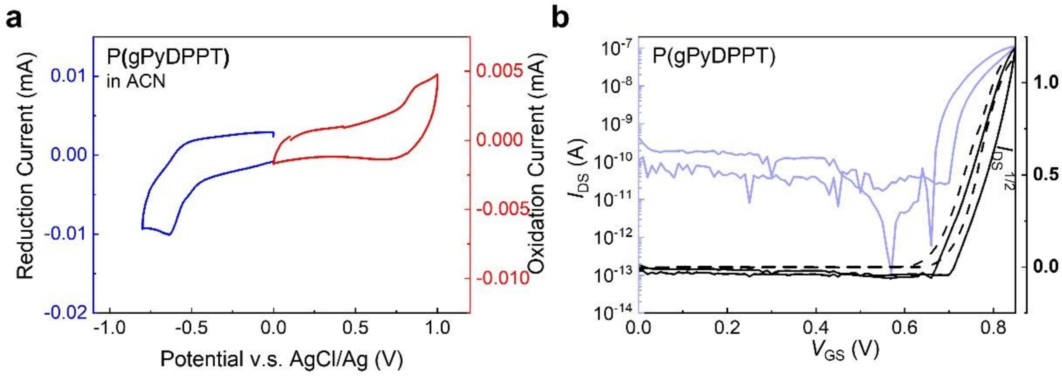

Fig. S11 Electrochemistry and device performance of P(gPyDPPT). a Cyclic voltammograms of P(gPyDPPT) in acetonitrile solution with 0.1 M tetrabutylammonium hexafluorophosphate as the electrolyte. **b** OECTs performance of P(gPyDPPT). Transfer characteristics of P(gPyDPPT). W/L = 100/10 µm and V_{DS} = 0.6 V. The μC^* is 0.07 F cm^{-1} V^{-1} s^{-1}.

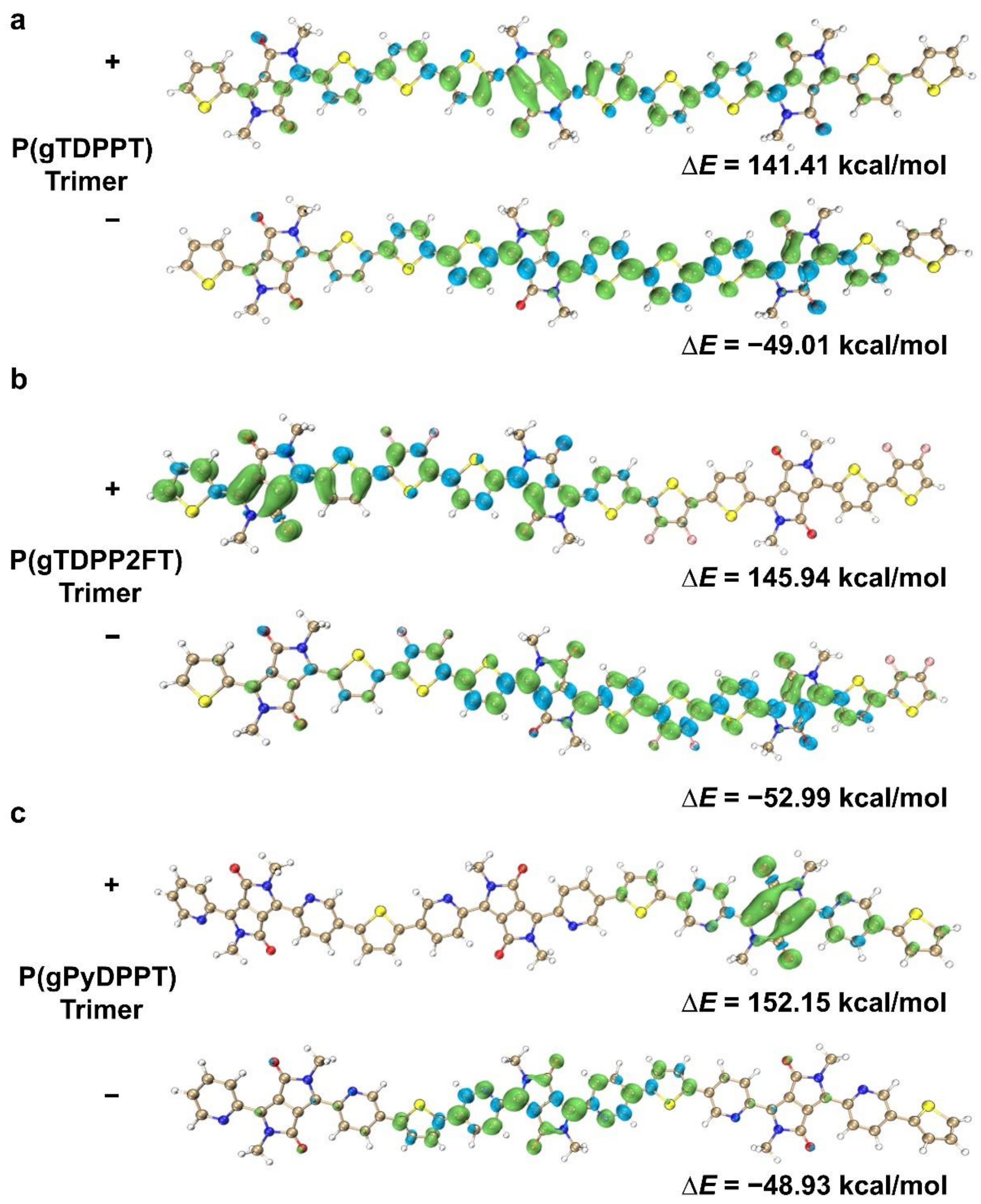

Fig. S12 Spin density distribution of positively (+) or negatively (−) charged polymers. a P(gTDPPT), **b** P(gTDPP2FT) and **c** P(gPyDPPT). Δ*E* stands for the electronic energy change from the neutral to the charged state. There is extra spin density on F atoms of negatively charged P(gTDPP2FT) compared to that of P(gTDPPT). The spin density delocalization length of positively charged P(gTDPPT) is the longest. Both the delocalization length of positively or negatively spin density for P(gPyDPPT) is the shortest.

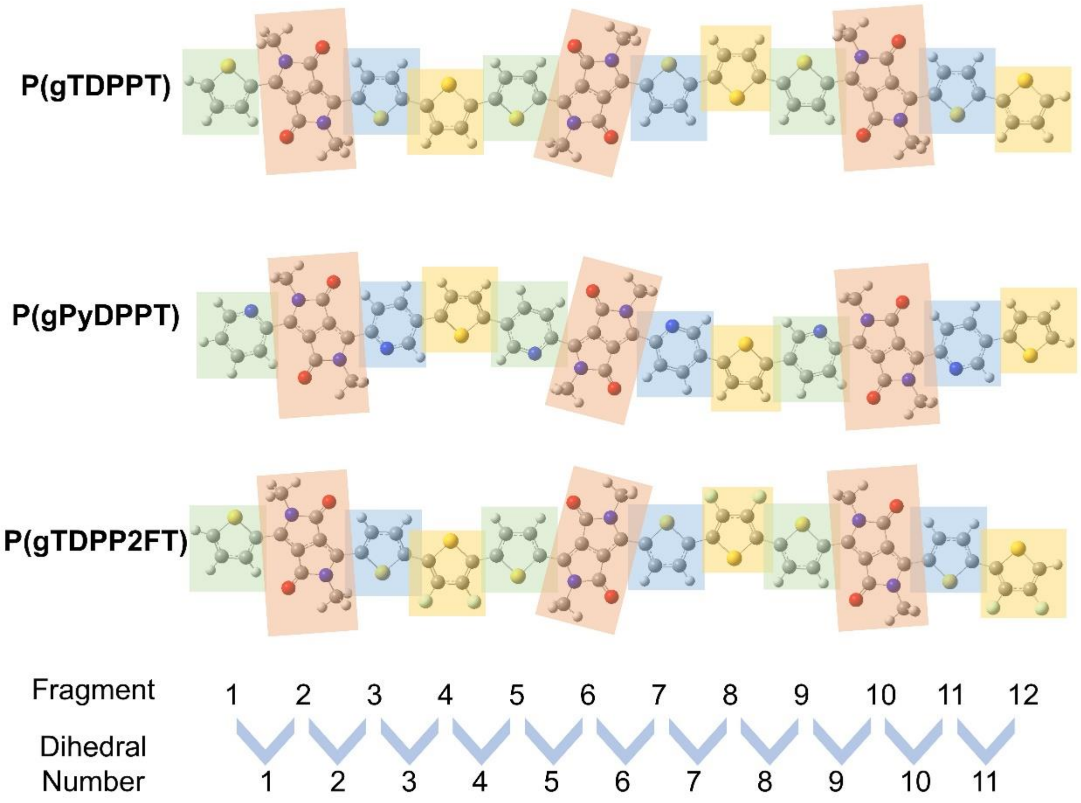

Fig. S13 Schematic diagram of fragment and dihedral numbers for the three polymers.

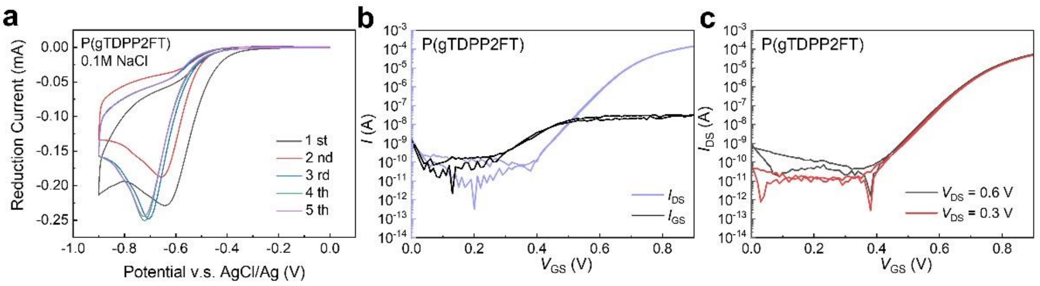

Fig. S14 Electrochemistry and device performance of P(gTDPP2FT). a Cyclic voltammograms of P(gTDPP2FT) in 0.1 M NaCl in air. The traps produced by O_2 can be exhausted during operation and the turn-on potentials shifted to the negative voltages. The low coulombic efficiency can be attributed to electrochemically irreversible of the ITO electrode. **b & c** Transfer characteristics of P(gTDPP2FT). *W/L* = 100/10 μm. The I_{GS} is four orders of magnitude lower than I_{DS} when the device is switched on. When the device is operated at low V_{DS} = 0.3 V, the I_{DS} is almost equal to that of V_{GS} = 0.6 V, consistent with the saturation range shown in the output curve. No parasitic side reactions were observed.

We observed oxygen reduction reaction (ORR) in the CV test (Fig. S14 a). The dissolved O_2 (much less than the partial pressure of oxygen in air) is reduced at the low potential in the first and second cycles, while the reduction peak is shifted to −0.7 V from the third cycle and remains stable. For OECT devices operating in highly-doped conditions, the traps produced by O_2 can be exhausted during operation and the devices can be switched on. Therefore, the impact of ORR on the device performance is limited, though the ORR will increase the threshold voltage (V_{th}) and decrease the charge carrier mobility. As for hydrogen evolution reactions, it is reported that most metal electrodes show hydrogen evolution potential larger than 1 V (−0.7 V vs SCE for Pt). Due to the low electrical conductivity and poor catalytic properties, semiconducting polymers have larger overpotentials. Therefore, no hydrogen evolution reaction was observed under the operation voltage of our devices.

3. Synthesis and Characterization of New Compounds

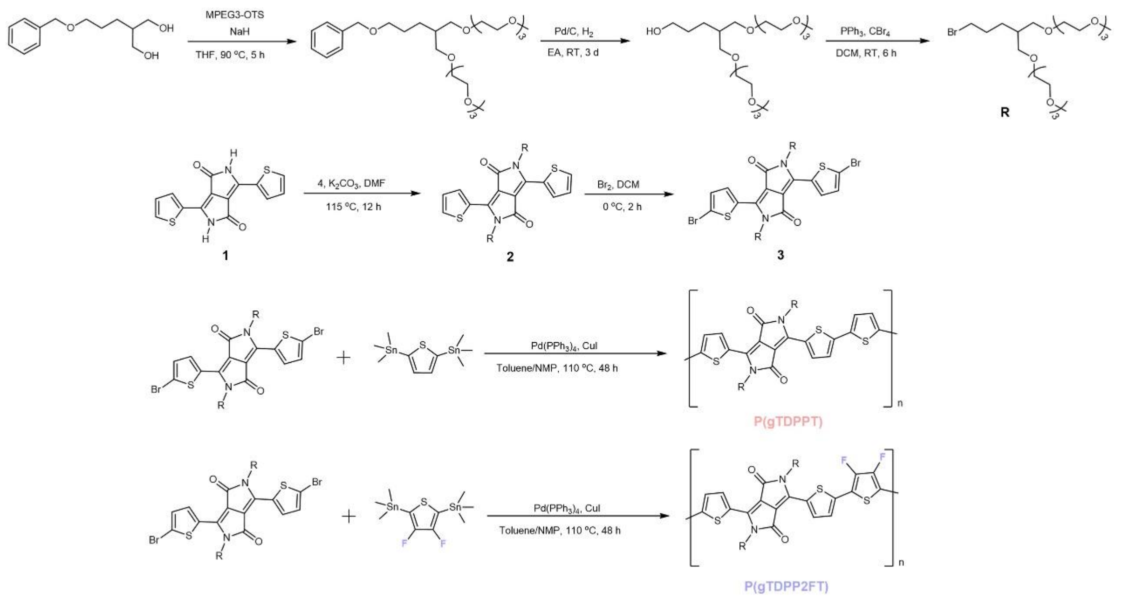

Fig. S15 Synthetic routes to the side chain R/monomer 3 and the polymers. The synthesis of the side chain R follows our previous work[9].

Synthesis of compound 2

Under nitrogen atmosphere, **1** (500 mg, 1.66 mmol) and potassium carbonate (1.14 g, 8.3 mmol) were added to 15 mL of *N, N*-dimethylformamide. After heating the mixture to 115 °C, **R** (1.6 g, 3.36 mmol) in DMF (5 mL) was added dropwise into the mixture and stirred for 12 h. After cooling to room temperature (RT), the mixture was washed with water and extracted with DCM. The residue was purified by silica gel column chromatography using EA/MeOH (*v:v, 30/1*) as the eluent to get a red solid **2** (350 mg, 31.3%). ^{1}H NMR ($CDCl_3$, 400 MHz, TMS), δ (ppm): 8.923-8.911 (d, J = 4.8, 2H), 7.681-7.666 (d, J = 6, 2 H), 7.301-7.279 (t, J = 8.8, 2H), 4.069-4.029 (t, J = 16 Hz, 4H), 3.642-3.594 (m, 34H), 3.542-3.527 (m, 16H), 3.423-3.396 (m, 6H), 3.369 (s, 12 H), 1.921-1.891 (m, 2H), 1.786-1.773 (m, 4H), 1.520-1.464 (m, 4H). ^{13}C NMR (100 MHz, CDCl3), d (ppm): 161.24, 139.92, 135.26, 130.94, 129.72, 128.64, 107.59, 71.93, 71.63, 70.64, 70.60, 70.52, 70.49, 59.04, 42.33, 38.80, 27.43, 25.75.

Synthesis of compound 3

2 (350 mg, 0.314 mmol) was added in 20 mL of DCM. After liquid bromine (102 mg, 0.643 mmol) was added slowly at 0 °C, the mixture was stirred for 2 h. After the reaction was quenched by $NaHSO_4$ aqueous solution, the mixture was washed with water and extracted with DCM. The residue was purified by silica gel column chromatography using EA/MeOH (*v:v, 40/1*) as the eluent to get a red solid **3** (205 mg, 51.25%). ^{1}H NMR ($CDCl_3$, 400 MHz, TMS), δ (ppm): 8.660-8.650 (d, J = 4, 2H), 7.266-7.256 (d, J = 4, 2H), 3.979-3.940 (t, J = 15.6, 4H), 3.644-3.601 (m, 34H), 3.565-3.529 (m, 16H), 3.447-3.382 (m, 6H), 3.372 (s, 12 H), 1.921-1.877 (m, 2H), 1.760-1.72 (m, 4H), 1.509-1.453 (m, 4H). ^{13}C NMR (100 MHz, CDCl3), d (ppm): 159.89, 137.85, 134.28, 130.71, 130.05, 118.16, 106.73, 70.92, 70.63, 69.63, 69.58, 69.51, 69.50, 69.47, 58.03, 41.39, 37.77, 26.49, 24.79. FTMS calcd. for $(M + H)^+$: 1274.36633, Found: 1274.40453.

Synthesis of polymer P(gTDPPT)

Tetratriphenylphosphine palladium (0.91 mg, 0.78 μmol), cuprous iodide (0.29 mg, 1.57 μmol), 2,5-bis(trimethylstannyl)thiophene (10.71 mg, 26.14 μmol) and **3** (34 mg, 26.66 μmol) and toluene/N-methylpyrrolidone (3 mL/3 mL) was added in a 25 mL Schlenk tube. The tube was charged with nitrogen through a freeze-pump-thaw cycle three times. The sealed tube was heated to 110 °C and stirred for 48 h. After cooling the reaction mixture to room temperature, diethylphenylazothioformamide (3 mg) was added to remove the catalyst and the resulting mixture was stirred at 80 °C for 1 h. The reaction mixture was poured into 50 mL hexane to precipitate and filter the polymer. The polymer solid was placed in a Soxhlet extractor and extracted with hexane, methanol, acetone, and chloroform. The chloroform solution was concentrated under reduced pressure and then poured into 20 mL hexane to reprecipitate the polymer P(gTDPPT). The suspension was filtered and dried in vacuum to afford the polymer. ^{1}H NMR ($CDCl_3$, 400 MHz, TMS), δ (ppm): 8.98, 7.42, 7.05, 5.32, 3.87, 3.63-3.54, 3.37, 1.98, 1.25, 0.88; M_n: 32.6 kDa; M_w: 67.4 kDa; PDI: 2.1. The P(gTDPPT) was

synthesized according to the literature[12].

Synthesis of polymer P(gTDPP2FT)

Tetratriphenylphosphine palladium (0.9 mg, 0.78 μmol), cuprous iodide (0.3 mg, 0.84 μmol), (3,4-difluorothiophene-2,5-diyl)bis(trimethylstannane) (11.58 mg, 25.98 μmol) and **3** (34.12 mg, 26.76 μmol) and toluene/N-methylpyrrolidone (3 mL/3 mL) was added in a 25 mL Schlenk tube. The tube was charged with nitrogen through a freeze-pump-thaw cycle three times. The sealed tube was heated to 110 °C and stirred for 48 h. After cooling the reaction mixture to room temperature, diethylphenylazothioformamide (3 mg) was added to remove the catalyst and the resulting mixture was stirred at 80 °C for 1 h. The reaction mixture was poured into 50 mL hexane to precipitate and filter the polymer. The polymer solid was placed in a Soxhlet extractor and extracted with hexane, methanol, acetone, and chloroform. The chloroform solution was concentrated under reduced pressure and then poured into 20 mL hexane to reprecipitate the polymer P(gTDPP2FT). The suspension was filtered and dried in vacuum to afford the polymer. ^{1}H NMR ($CDCl_3$, 400 MHz, TMS), δ (ppm): 9.07, 7.03, 3.76, 3.64-3.54, 3.37, 2.0, 1.25, 0.86. M_n: 30.7 kDa; M_w: 65.0 kDa; PDI: 2.1.

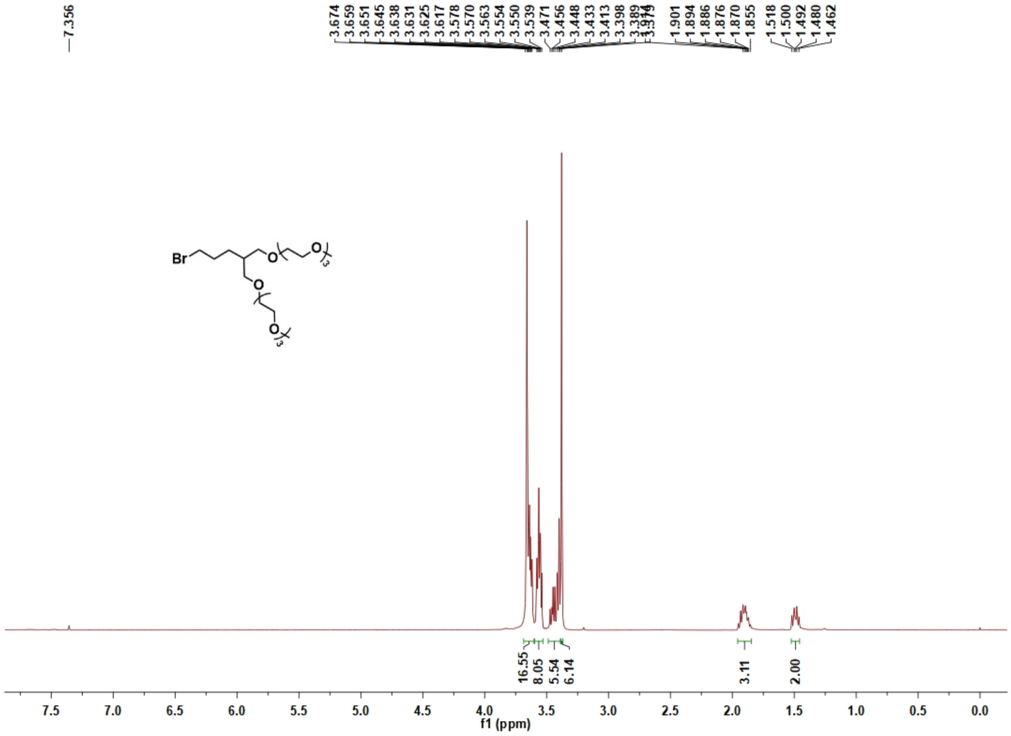

Fig. S16 ^{1}H NMR spectrum of compound R. The spectrum is collected using $CDCl_3$ as the solvent at 298 K.

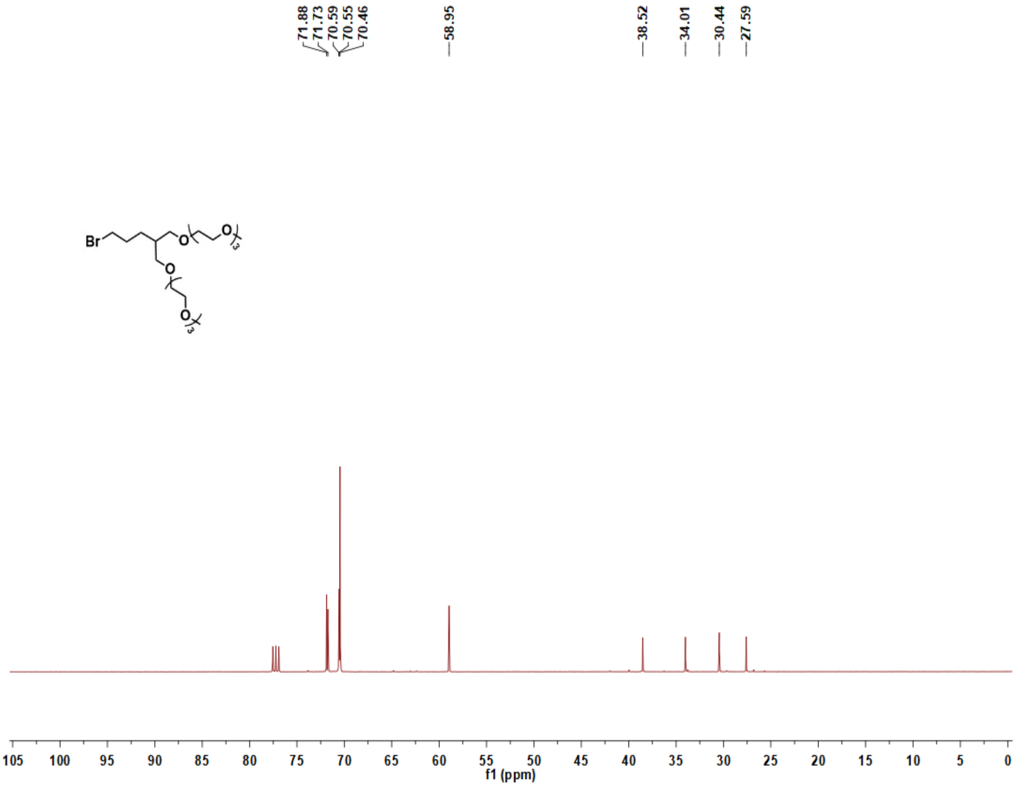

Fig. S17 ^{13}C NMR spectrum of compound R. The spectrum is collected using $CDCl_3$ as the solvent at 298 K.

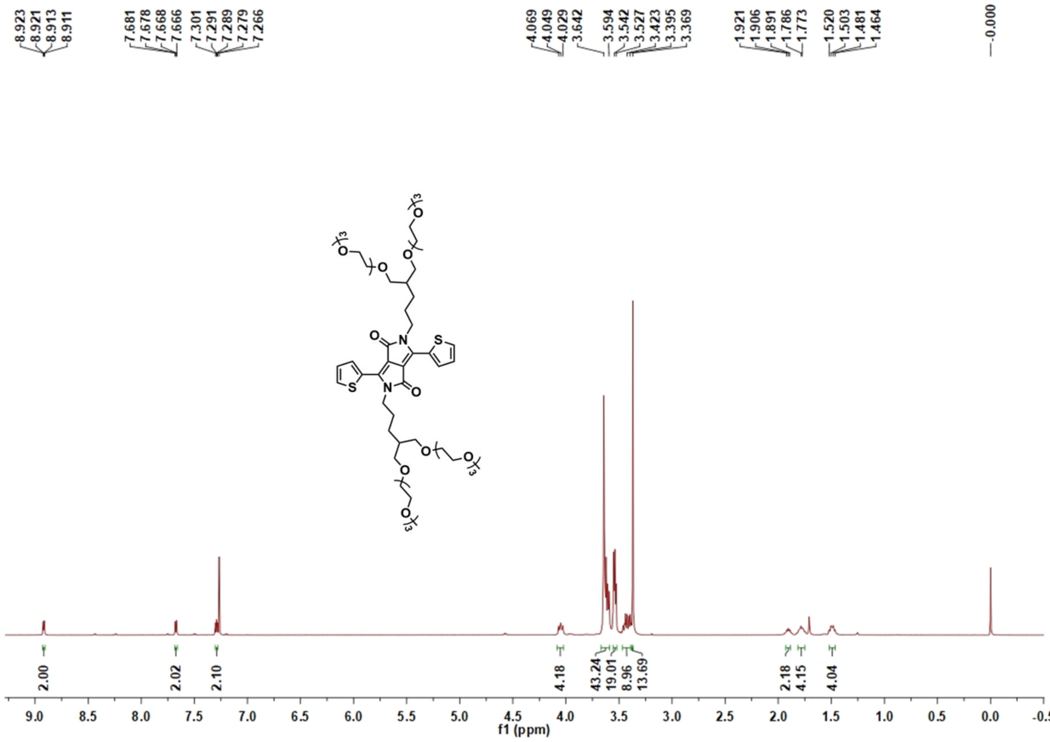

Fig. S18 ^{1}H NMR spectrum of compound 2. The spectrum is collected using $CDCl_3$ as the solvent at 298 K.

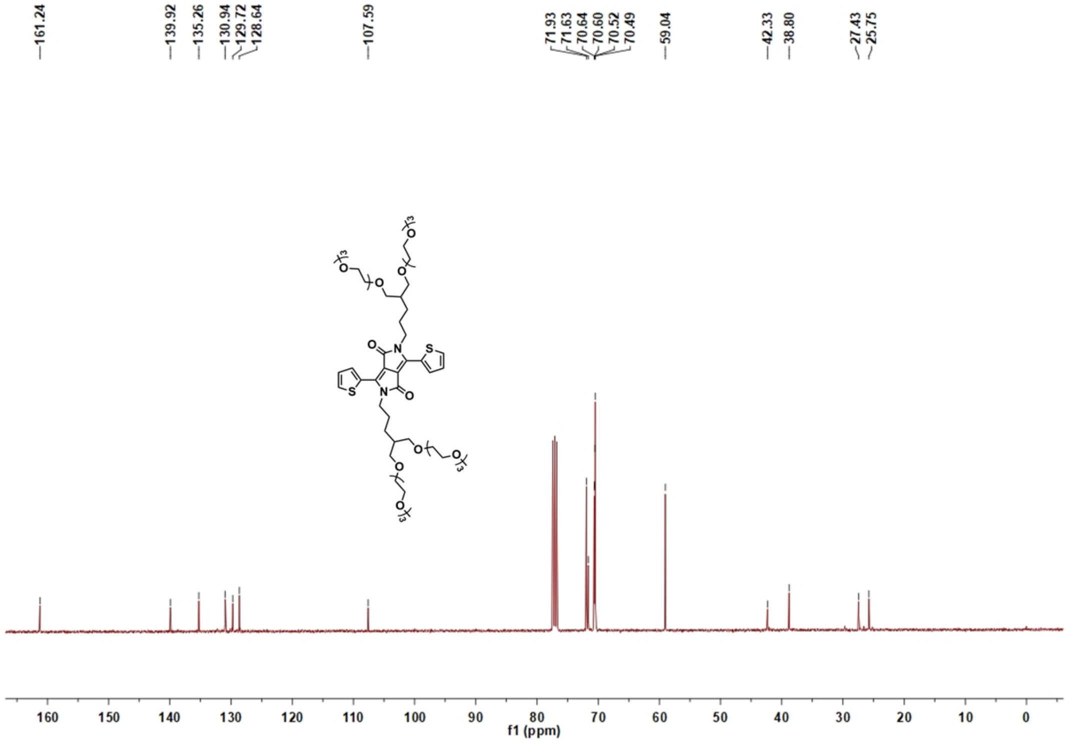

Fig. S19 ^{13}C NMR spectrum of compound 2. The spectrum is collected using $CDCl_3$ as the solvent at 298 K.

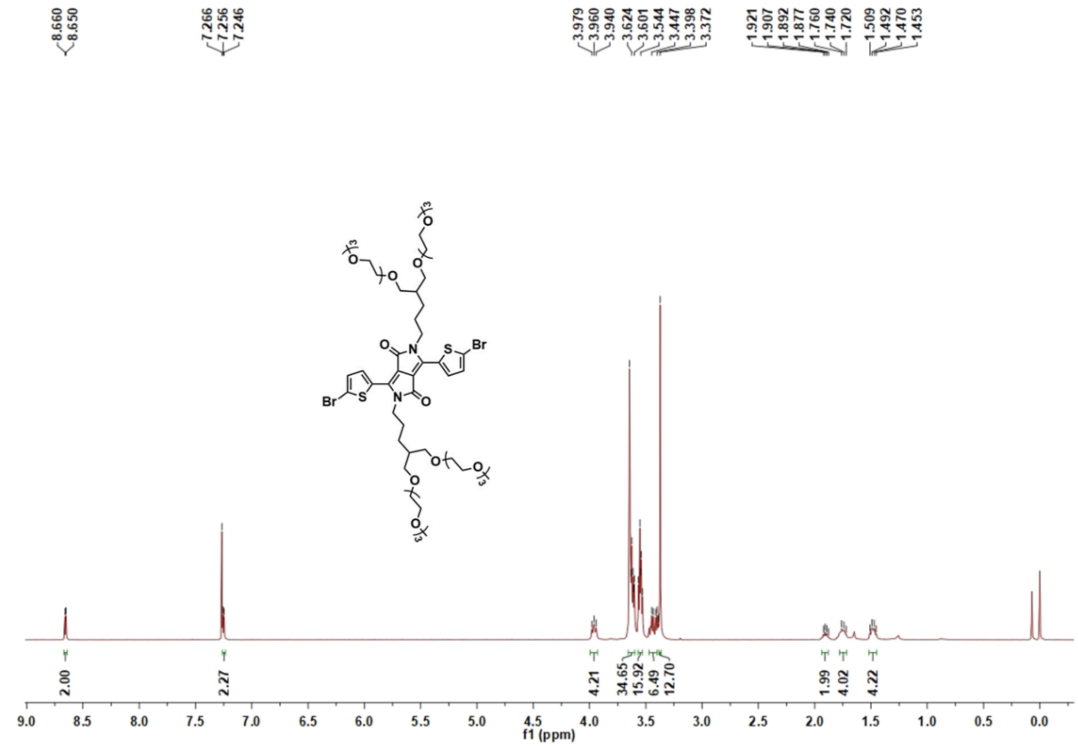

Fig. S20 ^{1}H NMR spectrum of compound 3. The spectrum is collected using $CDCl_3$ as the solvent at 298 K.

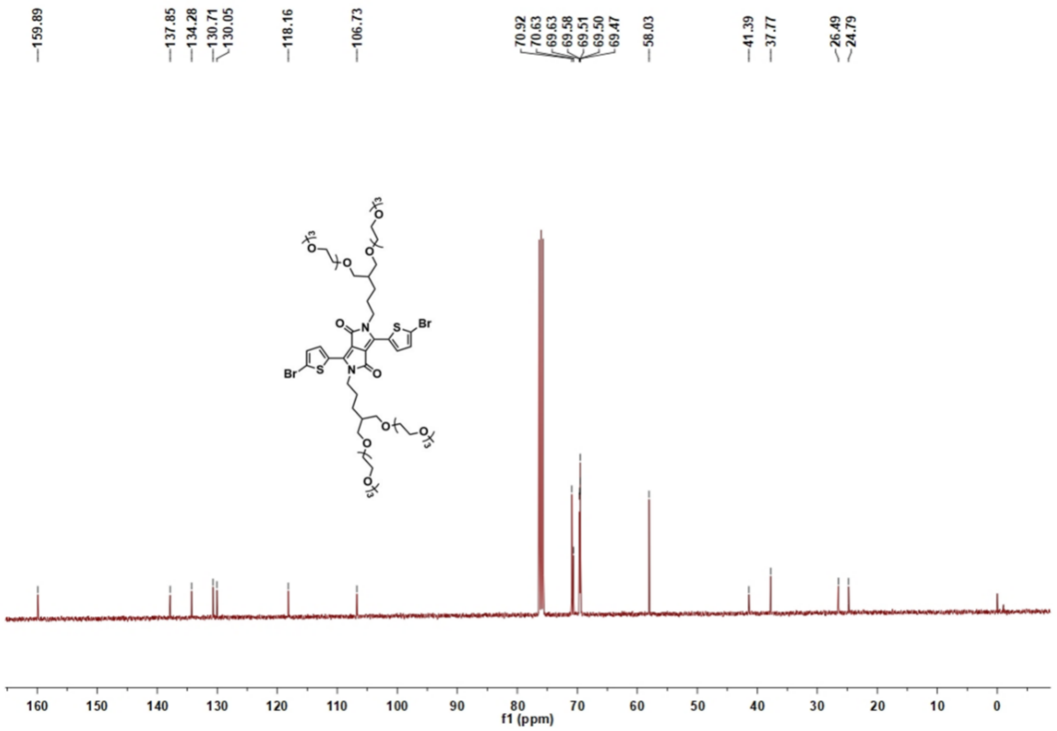

Fig. S21 ^{13}C NMR spectrum of compound 3. The spectrum is collected using $CDCl_3$ as the solvent at 298 K.

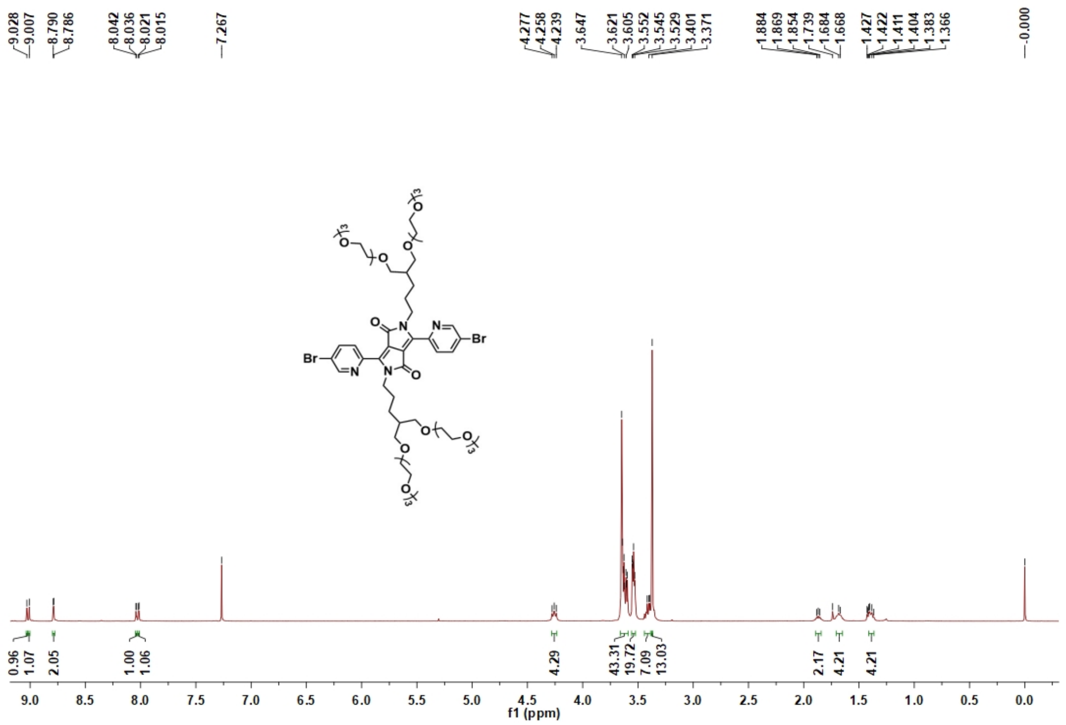

Fig. S22 ^{1}H NMR spectrum of compound gPyDPP. The spectrum is collected using $CDCl_3$ as the solvent at 298 K.

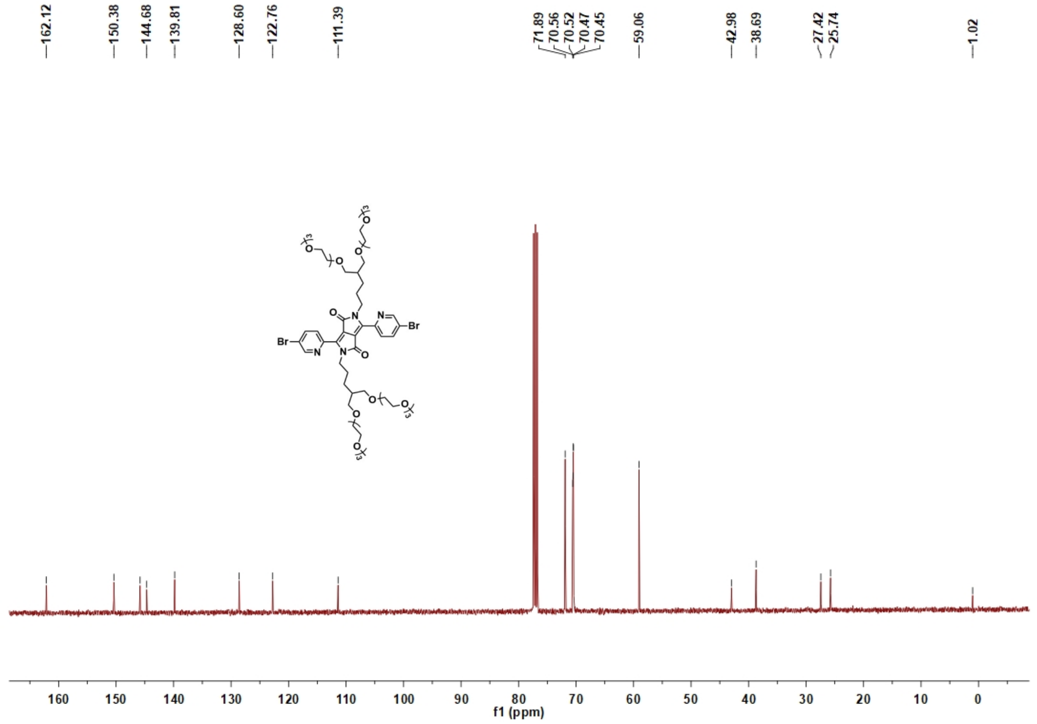

Fig. S23 ^{13}C NMR spectrum of compound gPyDPP. The spectrum is collected using $CDCl_3$ as the solvent at 298 K.

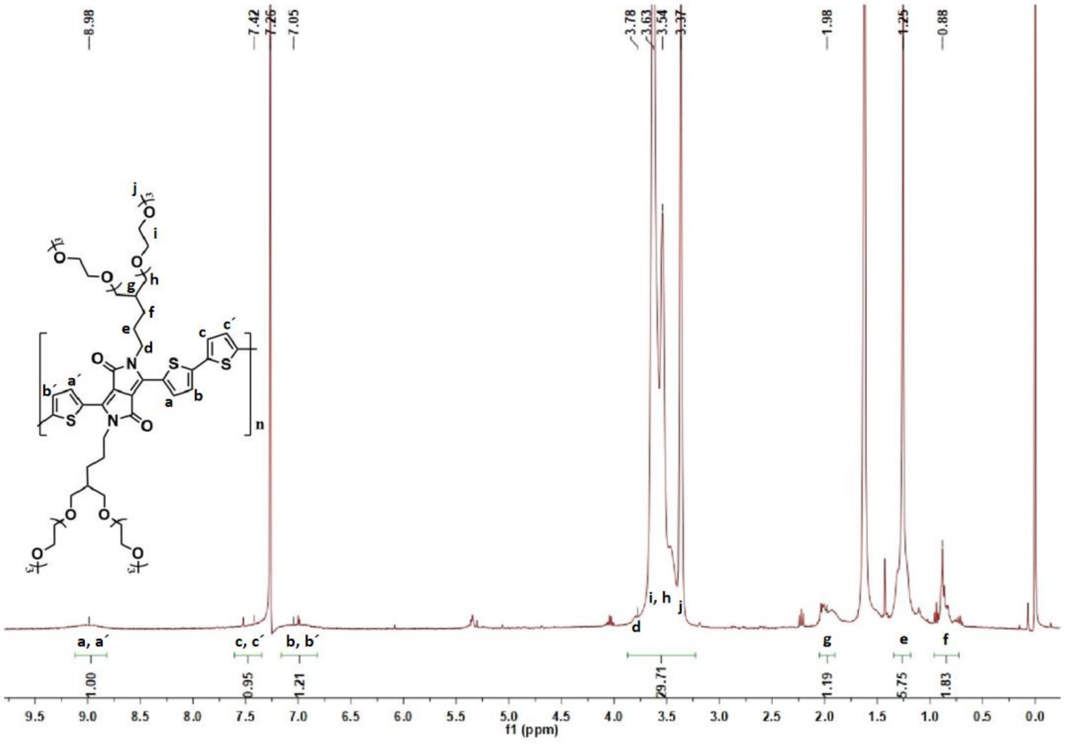

Fig. S24 ^{1}H NMR spectrum of polymer P(gTDPPT). The spectrum is collected using $CDCl_3$ as the solvent at 298 K.

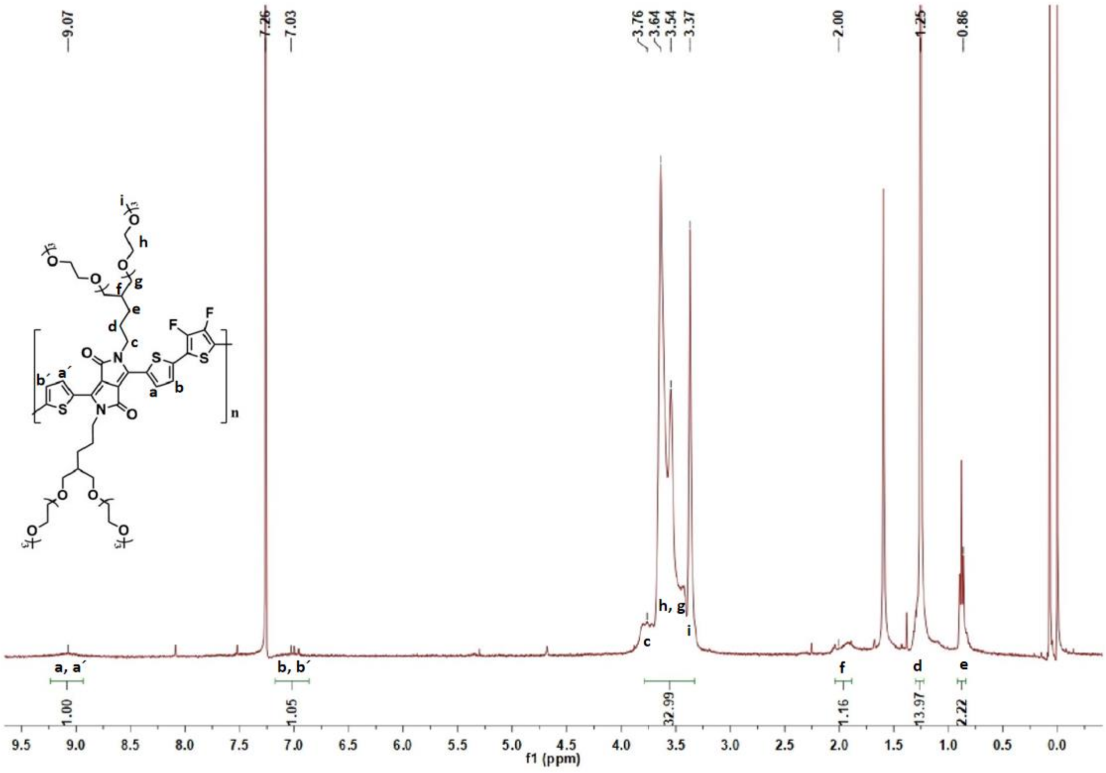

Fig. S25 ^{1}H NMR spectrum of polymer P(gTDPP2FT). The spectrum is collected using $CDCl_3$ as the solvent at 298 K.

Supplementary References

1. Frisch MJ*, et al.* Gaussian 16 rev. C.01. Wallingford, CT %! Gaussian 16; 2016.
2. GaussView V, Roy Dennington, Todd A. Keith, and John M. Millam, Semichem Inc., Shawnee Mission, KS, 2016.
3. Lu T, Chen F. Multiwfn: A multifunctional wavefunction analyzer. *J. Comput. Chem.* **33**, 580-592 (2012).
4. Humphrey W, Dalke A, Schulten K. Vmd: Visual molecular dynamics. *J Mol Graph* **14**, 33-38, 27-38 (1996).
5. Ohayon D*, et al.* Influence of side chains on the n-type organic electrochemical transistor performance. *ACS Appl. Mater. Interfaces* **13**, 4253-4266 (2021).
6. Wu HY*, et al.* Influence of molecular weight on the organic electrochemical transistor performance of ladder-type conjugated polymers. *Adv. Mater.* **34**, e2106235 (2022).
7. Feng K*, et al.* Fused bithiophene imide dimer-based n-type polymers for high-performance organic electrochemical transistors. *Angew. Chem. Int. Ed.* **60**, 24198-24205 (2021).
8. Wang YZ*, et al.* Green synthesis of lactone-based conjugated polymers for n-type organic electrochemical transistors. *Adv. Funct. Mater.* **32**, 2111439 (2022).
9. Shi JW*, et al.* Revealing the role of polaron distribution on the performance of n-type organic electrochemical transistors. *Chem. Mater.* **34**, 864-872 (2022).
10. Marks A*, et al.* Synthetic nuances to maximize n-type organic electrochemical transistor and thermoelectric performance in fused lactam polymers. *J. Am. Chem. Soc.* **144**, 4642-4656 (2022).
11. Feng K*, et al.* Cyano-functionalized n-type polymer with high electron mobility for high-performance organic electrochemical transistors. *Adv. Mater.* **34**, e2201340 (2022).
12. Jia H*, et al.* Engineering donor–acceptor conjugated polymers for high-performance and fast-response organic electrochemical transistors. *J. Mater. Chem. C* **9**, 4927-4934 (2021).
